# Comparative Effectiveness of Different Strategies of Oral Cholera Vaccination in Bangladesh: A Modeling Study

**DOI:** 10.1371/journal.pntd.0003343

**Published:** 2014-12-04

**Authors:** Dobromir T. Dimitrov, Christopher Troeger, M. Elizabeth Halloran, Ira M. Longini, Dennis L. Chao

**Affiliations:** 1 Center for Statistics and Quantitative Infectious Diseases; Vaccine and Infectious Disease Division; Fred Hutchinson Cancer Research Center; Seattle, Washington, United States of America; 2 Department of Biostatistics; University of Washington; Seattle, Washington, United States of America; 3 Department of Biostatistics; University of Florida; Gainesville, Florida, United States of America; Oxford University Clinical Research Unit, Vietnam

## Abstract

**Background:**

Killed, oral cholera vaccines have proven safe and effective, and several large-scale mass cholera vaccination efforts have demonstrated the feasibility of widespread deployment. This study uses a mathematical model of cholera transmission in Bangladesh to examine the effectiveness of potential vaccination strategies.

**Methods & Findings:**

We developed an age-structured mathematical model of cholera transmission and calibrated it to reproduce the dynamics of cholera in Matlab, Bangladesh. We used the model to predict the effectiveness of different cholera vaccination strategies over a period of 20 years. We explored vaccination programs that targeted one of three increasingly focused age groups (the entire vaccine-eligible population of age one year and older, children of ages 1 to 14 years, or preschoolers of ages 1 to 4 years) and that could occur either as campaigns recurring every five years or as continuous ongoing vaccination efforts. Our modeling results suggest that vaccinating 70% of the population would avert 90% of cholera cases in the first year but that campaign and continuous vaccination strategies differ in effectiveness over 20 years. Maintaining 70% coverage of the population would be sufficient to prevent sustained transmission of endemic cholera in Matlab, while vaccinating periodically every five years is less effective. Selectively vaccinating children 1–14 years old would prevent the most cholera cases per vaccine administered in both campaign and continuous strategies.

**Conclusions:**

We conclude that continuous mass vaccination would be more effective against endemic cholera than periodic campaigns. Vaccinating children averts more cases per dose than vaccinating all age groups, although vaccinating only children is unlikely to control endemic cholera in Bangladesh. Careful consideration must be made before generalizing these results to other regions.

## Author Summary

Bangladesh has a high burden of cholera and may become the first country to use cholera vaccine on a large scale. Mass cholera vaccination may be hard to justify to international funding agencies because of the modest efficacy of existing vaccines and their limited duration of protection. However, mass cholera vaccination can induce high levels of indirect protection in a population, i.e., protecting even unvaccinated individuals by lowering cholera incidence, and a case for cost-effective cholera vaccination could be made. Mathematical modeling is one way to predict the magnitude of indirect protection conferred by a proposed vaccination program. Here, we predict the effectiveness of various mass cholera vaccination strategies in Bangladesh using a mathematical model. We found that maintaining high levels of vaccination coverage in children could be very effective in reducing the burden of cholera, and secondary transmission of cholera would virtually stop when 70% of the population is vaccinated. Mathematical modeling may play a key role in planning widespread cholera vaccination efforts in Bangladesh and other countries.

## Introduction

*Vibrio cholerae*, the bacterium responsible for clinical cholera, has long been associated with the Bay of Bengal where it exists as an autochthonous member of the estuarine ecosystem [1], [2]. This area of south Asia has been the origin for six of the seven cholera pandemics, and the burden of disease remains high. Today cholera is endemic in much of the Ganges River Delta with an estimated 350,000 treated cases per year in Bangladesh alone [3]. Improvements in water, sanitation, and hygiene are the long-term solutions for cholera, but oral cholera vaccines (OCVs) may constitute a shorter-term option to reduce morbidity and mortality from the disease.

Oral cholera vaccines are safe and effective [4]–[6]. A recent large field trial in Kolkata, India, has shown that *Shanchol*, one of two World Health Organization prequalified OCVs, provides 65% protection over 5 years [5]. Further, successful demonstration campaigns conducted by the International Centre for Diarrhoeal Disease Research, Bangladesh (icddr,b) in urban and rural communities show promise for expanding vaccination coverage in Bangladesh [4], [7]. The expanded use of OCV as a component of cholera control is supported by the recent decision by the World Health Organization to establish a global stockpile of 2 million doses of OCV, and the GAVI Alliance has committed to finance and leverage support for the global stockpile through 2018 [8], [9]. Although OCV will likely be more widely used in the coming years, its effectiveness at a population level is not well understood. This information is crucial for planning vaccination programs on a large scale.

It can be difficult to justify the widespread use of OCVs on economic grounds because: OCVs confer only moderate protection for a few years [5], [10], the incidence of cholera in most settings is relatively low, and the number of deaths attributed to cholera is relatively small because of the availability of inexpensive and effective treatment. However, large vaccine trials have shown that as OCV coverage increases, indirect protection from vaccination, also known as herd protection, increases [11], [12]. When indirect protection is considered, the effectiveness of mass cholera vaccination can be high, and accounting for the effects of indirect protection appears to be necessary to make OCVs cost effective in the developing world [3], [13]–[15]. Mathematical models can be used to predict the effectiveness of mass cholera vaccination, including indirect effects [16]–[20], so modeling may be an essential component of any economic case for cholera vaccination. An earlier modeling study found that vaccinating 50 to 70% of the population of Bangladesh would virtually eliminate transmission [16]. Here, we expand upon that work, using a mathematical model to predict the effectiveness of targeting different age cohorts for vaccination at various coverage levels and schedules over a 20-year horizon.

## Methods

### Data

Matlab is a rural community of approximately 220,000 people 30 kilometers southeast of Dhaka [21]. Data on cholera cases in Matlab were collected during long-term passive surveillance as described in detail elsewhere [22]. Briefly, between 1997 and 2001, twice a month for three days a study physician attended to all patients presenting with acute watery diarrhea at the icddr,b clinic. Following orally obtained informed consent, these patients were tested for *V. cholerae* by rectal swab, and cultured on the same day in the Matlab laboratory [22], [23]. Patient data, including age and sex, were obtained with identifying information removed. The Committee on Human Research of the Johns Hopkins University Bloomberg School of Public Health approved the research, and its guidelines were followed in the conduct of the clinical research.

### Mathematical model

**Figure 1:**
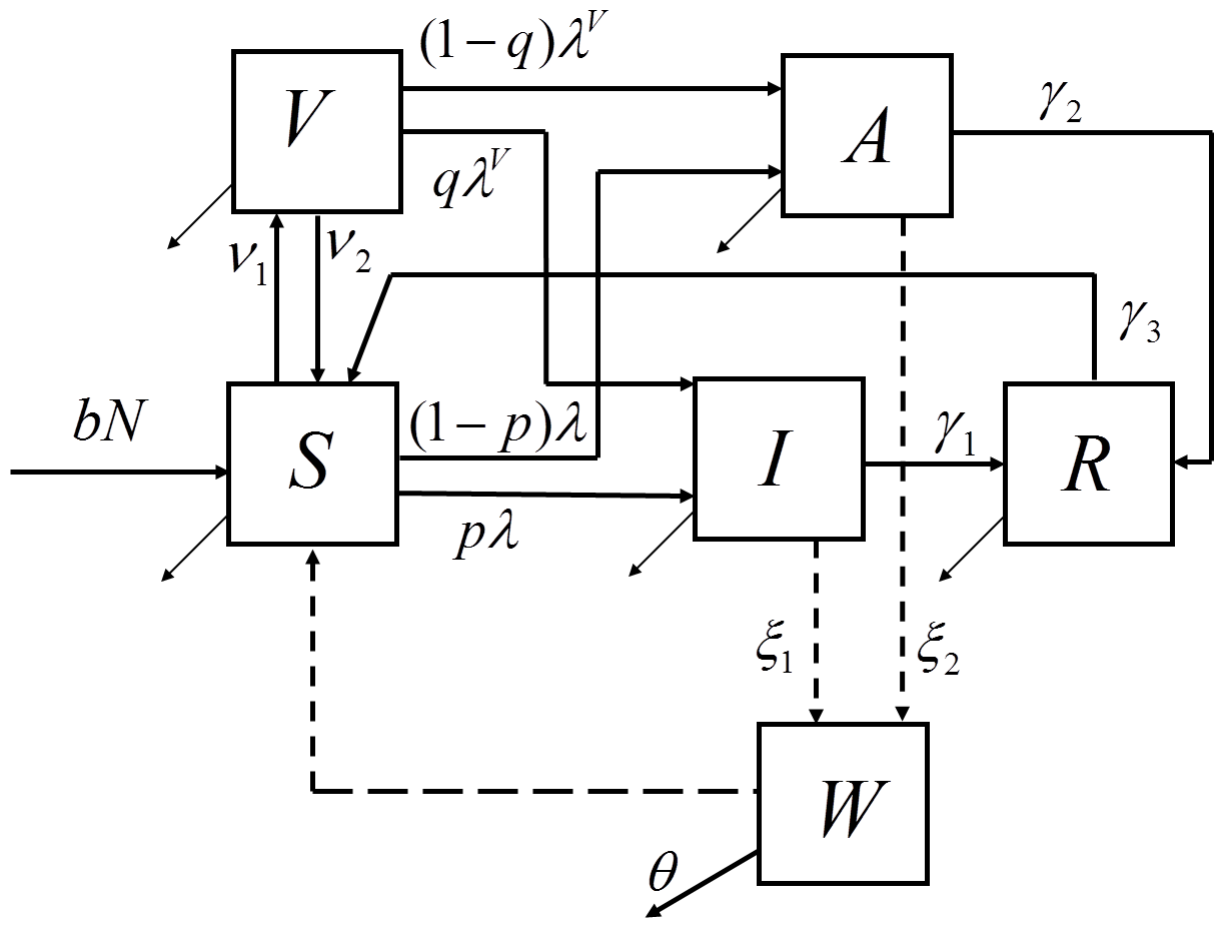
Flow diagram of the mathematical model of cholera transmission. Individuals are aggregated in compartments by cholera status as susceptible unvaccinated (S), susceptible vaccinated (V), cholera cases (I), asymptomatically infected (A), and recovered (R). The concentration of *Vibrio cholerae* in the environment is tracked in a separate compartment (W). Proportion *p* of the susceptibles and proportion *q* of the vaccinated who become infected are symptomatic (cholera cases), and a fraction *r* of these are reported and used to fit the model to surveillance data. Infected individuals shed bacteria into the environment and cause indirect transmission of Cholera (dashed lines). The simulated population is additionally stratified by age (not shown). A complete description of the model including the expressions for the forces of infections (λ and λ^V^) is presented in Text S1.

We developed an age-structured mathematical model of cholera transmission. Compartments in the model are unvaccinated susceptible (S), vaccinated susceptible (V), symptomatically infected (I), asymptomatically infected (A), or recovered and immune (R) from cholera (Figure 1). The concentration of *V. cholerae* in the environment (water) is tracked in an additional compartment (W). Susceptible individuals may become infected by direct contact with infected individuals (direct transmission) or by exposure to *V. cholerae* in the environment (indirect transmission). A complete description of the model is given in Text S1.

The model aggregates the population in compartments by disease status and age. Age cohorts represent children under 2 years old, pre-school aged children (2 to 4 years old), school aged children (5 to 14 years old), and adults (15 years old and older). Younger age groups are assumed to be more susceptible to infection [24]. Births are modeled by adding unvaccinated susceptibles to the youngest age cohort each year, and deaths are modeled by removing individuals from all age cohorts. Birth and age-specific mortality rates were based on data from the Matlab Health and Demographic Surveillance System [21]. Cohorts are aged by moving individuals into the next older age compartment at the appropriate rates.

Frequency-dependent transmission rates are assumed for infections acquired through short cycle transmission (person-to-person) while a saturation (Holling type II [25]) function in terms of cholera concentration in water (W) is used to model the force of infection from long cycle transmission (environmental exposure). A fraction *p* of the infections are symptomatic, a fraction *r* of which seek treatment. We refer to *r* as the reporting rate. The asymptomatically infected individuals (proportion *1-p* of all infections) are less infectious and shed bacteria into the environment at a lower rate than cholera cases. Throughout this paper, “cholera cases” refers to the number of symptomatically infected individuals. The reporting rate of cholera cases is set to 10% in the main scenario and to 25% in alternative scenarios to test the sensitivity of results to this parameter [26].

Infected individuals recover after five days on average and are immune to infection until they transition back to the susceptible state after an average of 3 years [27], [28]. An alternative scenario, assuming different duration of natural immunity protection across age groups, is also investigated.

**Figure 2:**
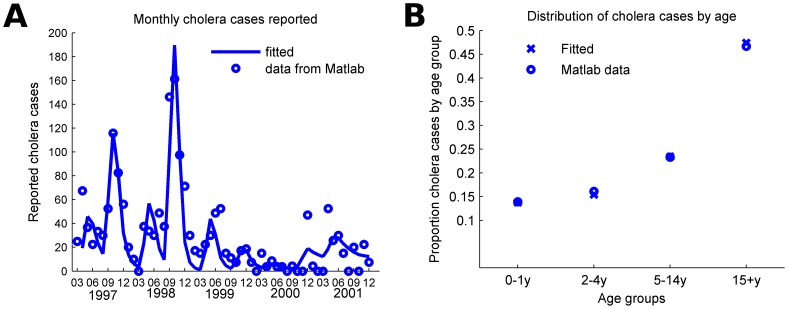
The mathematical model was calibrated to fit both the seasonal dynamics and the age distribution of cases in Matlab, Bangladesh. A) Reported cholera cases per month in Matlab, Bangladesh from March 1997 to December 2001. B) Distribution of the reported cholera during this period by age group.

The model is calibrated to fit the dynamics of cholera cases recorded between 1997 and 2001 in Matlab. The proportion of recovered individuals at the beginning of 1997 is estimated based on time-series data of cholera incidence in Matlab [29], the assumed reporting rate, and the duration of natural immunity. Two periods of increased environmental transmission occur annually with the first peak occurring in spring (approximately April to May) followed by a larger peak in autumn (approximately September to November) [30], [31]. An iterative fitting procedure is implemented in which one cholera season (1997–1998) is simulated to estimate: i) the initial distribution of the recovered individuals by age groups by running the model for 5 years and rescaling back to the estimated overall recovered proportion; ii) the transmission rates for both short and long cycle transmission by fitting the number of monthly symptomatic infections based on data for reported cases; and iii) the relative susceptibility of each age group by fitting the observed age distribution of cholera cases. Next, the estimated transmission rates are used to estimate the magnitude of elevated environmental risk during spring and autumn periods as well as the start time of the autumn period by fitting the monthly cases reported between 1998 and 2001. The resulting best fit of the dynamics, minimizing the residual sum of squares for the number of reported cases per month, are presented in Figure 2. Parameters used in the model are presented in Table S1 in Text S2 and the values of the estimated parameters are presented in Tables S2 & S3 in Text S2. The magnitude of the elevated environmental risk during peak periods is sampled from the aggregated ranges in Table S3 in Text S2, which represent the variation fitted over 5 consecutive annual cycles.

Vaccinated susceptibles in the model are protected for an average of five years. Adults and children 5 years and older are 65% less likely to become infected upon exposure to cholera than unvaccinated individuals and children 1–4 years are 40% less likely to become infected [5]. OCVs may decrease the probability of developing symptoms upon infection [32], but insufficient trial data is available to include this effect in the model. Therefore, we took a conservative approach and assumed that upon infection, vaccinated individuals have the same probability of becoming symptomatic as and are as infectious as non-vaccinated individuals. The model was implemented in Matlab R2012a (The MathWorks, Inc.).

### Modeling cholera vaccination strategies

We modeled vaccination programs that target one of three age groups: the entire vaccine-eligible population (those one year old and older), all children (ages one to fourteen years), and preschoolers (ages one to four years). We did not model the vaccination of those under one year old, since no vaccine is currently licensed for that age group [33]. The age structure of the model does not precisely match the age cohorts targeted for vaccination. The age cohorts in the model were chosen to match the age groups in the available epidemiological data for calibration. The age cohorts for vaccination were chosen to match current vaccine licensing and logistical considerations. Vaccination of one-year-olds in all scenarios is modeled by targeting half of the population younger than 2 years old. If cholera vaccines are later licensed for use in infants (i.e., under one year old), one could vaccinate a larger fraction of the youngest age cohort in the model.

We modeled three distinct schedules for vaccinating these target populations: one-time campaign, periodic campaigns, and continuous vaccination. For the one-time campaign, a proportion of the targeted population is vaccinated at the start of the first year only. For the periodic campaigns, every five years a proportion of all susceptible and recovered individuals are vaccinated. The period between campaigns was chosen to match the duration of vaccine protection. The continuous vaccination strategy is an approximation of an annual vaccination program. In this strategy, a proportion of the targeted population is vaccinated at the beginning of the first year, then starting in the second year the unvaccinated susceptible and recovered individuals are vaccinated at a fixed rate for the duration of the simulation. A detailed description of the implementations of all vaccination strategies in the model is in Text S1.

We define the overall effectiveness of mass vaccination to be the number of cholera cases prevented (i.e., the difference in the number of cases in a simulation without vaccination and the number of cases in a simulation with mass vaccination) divided by the number of cases when there is no vaccination [34]. We measure the efficiency of a mass vaccination strategy by the number of vaccinations per case averted (VPC), calculated as the number of people who are vaccinated divided by the number of cholera cases prevented.

## Results

Seasonal cholera transmission was simulated in a rural population in Bangladesh using a mathematical model calibrated to reproduce the two annual peaks (Figures 2A and S1) and the age distribution of cases observed in surveillance from the community of Matlab (Figure 2B). We found that infants and children younger than two years old, preschoolers (ages 2 to 4 years) and school children (ages 5 to 14 years) were 6.3, 5.2, and 1.8 times more susceptible than adults, respectively, to best fit the data assuming the same duration of immunity after infection across ages (Table S2 and Figure S2 in Text S2). We also estimated that asymptomatically infected individuals were 15% as infectious as the symptomatic cholera cases assuming that 20% of all infected individuals become symptomatic and 10% of cholera cases are reported.

**Figure 3:**
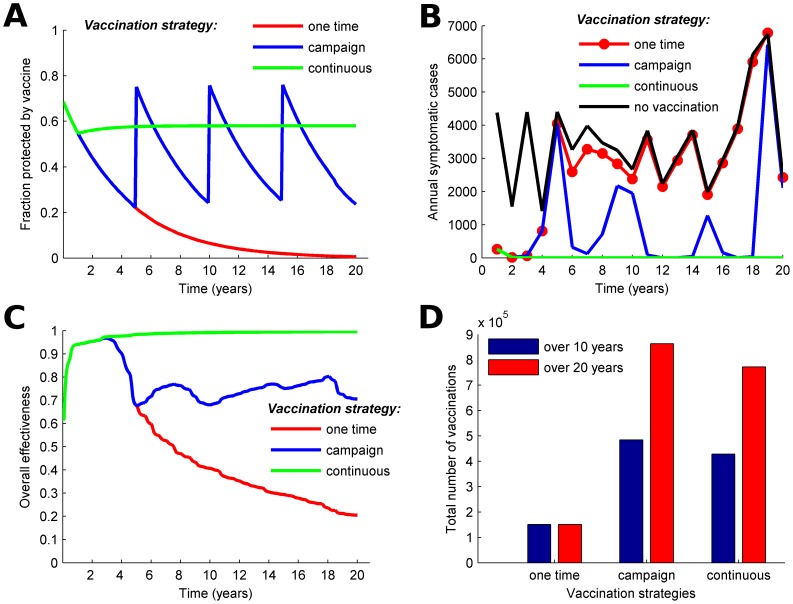
Comparison of one-time mass vaccination, vaccination campaigns that occur every 5 years, and continuous vaccination. The one-time vaccination targets 70% of the vaccine-eligible population (those one year old and older) for vaccination, the 5-year campaigns target 70% of the population every 5 years, and the continuous vaccination strategy targets 70% of the population at the beginning of the first year then vaccinates at a constant rate starting in year 2. Vaccine is less effective for children aged 1–4 (40% efficacy) than for older children and adults (65% efficacy). Temporal dynamics of A) the fraction of the population protected by vaccine; B) annual cholera cases; C) the overall effectiveness in terms of prevented fraction of cholera cases when different vaccination strategies are implemented; and D) total number of vaccinations for different strategies over 10 and 20 years.

We compare the effectiveness over 20 years of one-time mass vaccination, recurring campaigns every five years, and continuous vaccination targeting 70% of all individuals one year old and older (Figure 3). All three vaccination strategies avert about 94% of the cholera cases in the first year (Figures 3B and 3C). Vaccination of 50% of the population would reduce the incidence of cholera by 88% in the first year following vaccination (Figure S3 in Text S2). This is consistent with projections from a previous modeling study that found vaccination coverage of 50% would be sufficient to avert 93% of cholera cases in one season in Matlab [16]. With a one-time mass vaccination campaign, cholera incidence rebounds as protection from vaccine wanes and new susceptible individuals are born, and the overall effectiveness of the campaign is only about 20% after 20 years (Figure 3C).

Because protection conferred by vaccination lasts five years, one might choose to conduct campaigns once every five years for logistical reasons. However, susceptibility in the population accumulates between campaigns and the proportion of the population protected by vaccine drops to 20%–25% due to the waning of vaccine efficacy and the birth of new susceptible individuals (Figure 3A). Vaccination campaigns every five years could result in 70% overall effectiveness over 20 years but cholera incidence oscillates and peaks in the years preceding each campaign (Figure 3B).

To avoid the fluctuations in vaccination coverage associated with 5-year campaigns, we modeled continuous vaccination in which people are vaccinated at a constant rate throughout the year every year after year 1. When calibrated to use nearly the same amount of vaccine as the 5-year campaigns (Figure 3D), 58% of the population is always protected by vaccine (Figure 3A). When the population is continuously vaccinated, cholera incidence remains low over the 20 years with overall effectiveness above 95% (Figure 3C), and onward cholera transmission is essentially interrupted after ten years. The continuous strategy achieves 25% higher overall effectiveness than the 5-year campaigns (Figure 3C) while using slightly less vaccine over 20 years (Figure 3D).

**Figure 4:**
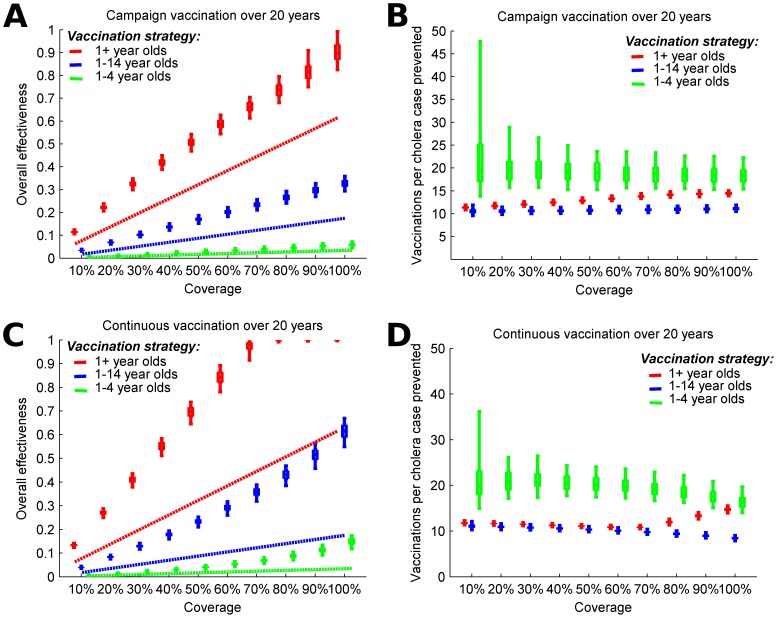
Modeling 5-year campaign and continuous vaccination strategies for vaccine with different coverage levels and targeting different age groups. A) Overall effectiveness and B) vaccinations per case prevented by mass vaccination campaigns that occur every 5 years. C) Overall effectiveness and D) vaccinations per case prevented by continuous vaccination. We assume that the vaccine protects for an average of 5 years and is less effective among children from ages 1 to 4 years (40% efficacy) than among older individuals (65% efficacy). Overall effectiveness is the prevented fraction of cholera cases over 20 years. The boxes represent the interquartile range and the whiskers cover 90% of the results from 100 simulations per scenario with parameters of seasonal environmental exposure sampled from ranges in Table S3. Dashed lines show the effectiveness if only direct effects of the vaccine are considered.

We compared the effectiveness of targeting different age groups with campaigns every five years. Our modeling results suggest that vaccinating everyone (100% of) one year old and older at 5-year intervals would prevent 89% of cholera cases over 20 years (Figure 4A, red boxes). The efficiency of the 5-year campaigns decreases with higher coverage, with the number of vaccinations per case averted (VPC) rising from 11 to 14 (Figure 4B). Mass vaccination of all children 1 to 14 years old at 5-year intervals would prevent approximately 33% of cholera cases (Figure 4A, blue boxes) while vaccinating all preschoolers would prevent only 6% of cholera cases over 20 years (Figure 4A, green boxes). Because the proportion of the population protected by vaccine drops between campaigns, this vaccination strategy is not able to suppress cholera activity over 20 years, even at 100% coverage (Figure 4A). Targeting children (1 to 14 years old) is most efficient; requiring about 11 VPC over a wide range of vaccination coverage levels (Figure 4B). Targeting those 1 to 4 years old is less efficient, primarily because of the lower vaccine efficacy in this group.

Continuous vaccination is associated with higher overall effectiveness than the 5-year campaigns. Coverage above 70% of the general population is sufficient to virtually interrupt onward transmission of cholera (Figure 4C). VPC associated with the continuous vaccination declines with increasing coverage when children (1 to 14 years old) are targeted. However, VPC increases, thus efficiency decreases, for coverage over 70% when the general population is vaccinated because effectively all cholera transmission is prevented above this level (Figure 4D).

If vaccine efficacy in young children were as high as that in adults, then children ages 1 to 4 years old would be the most efficient age group to target, and vaccinating 70% of them every 5 years would have a VPC of 7, and maintaining 70% coverage with continuous vaccination would have a VPC of 6.5 (Figure S4 in Text S2). If the entire vaccine-eligible population were targeted, then this vaccine would be associated with only a modest increase in overall effectiveness compared to the vaccine with lower efficacy in young children (Figure S4 in Text S2). If the vaccine confers protection for only 3 years instead of 5 and has 65% efficacy among all age groups, one could achieve effectiveness similar to the 5-year campaigns described above by vaccinating every three years, but more vaccine would be required (Figure S5 in Text S2).

Simulated cholera epidemics are sensitive to the assumed proportion of cases that seek treatment. An alternative scenario was calibrated assuming 25% of cholera cases seek treatment in Matlab, resulting in a lower underlying disease burden than the main analysis, which assumed a 10% reporting rate. This alternative scenario projects substantially smaller epidemics and consequently stronger impact of all vaccination programs (Figures S6 and S7 in Text S2). Approximately 30% coverage was enough for the 5-year campaigns and continuous vaccination to eliminate 90% of cholera cases. The same reduction is achieved by campaign vaccination of 80% or continuous vaccination of 60% of children (1 to 14 years old). However, the projected recovered fractions (Figure S6B in Text S2) for all age groups are substantially lower compared to the extrapolations based on the Matlab data (Figure S2 in Text S2), which argues against the plausibility of this high reporting rate. We also modeled an alternative scenario in which children are protected against cholera for a shorter time than adults after infection. In this model, the fraction of susceptible individuals in each age group differed from those seen when all individuals become susceptible an average of three years after infection, and mass vaccination was somewhat more effective (Figures S8–S9 in Text S2).

## Discussion

We used a mathematical model to explore the potential effectiveness of mass cholera vaccination in rural Bangladesh and believe that the results apply more broadly to cholera endemic areas in Bangladesh. With the model, we were able to predict the overall effectiveness, which includes indirect effects, of different mass vaccination strategies. Our results indicate that maintaining 60% or higher vaccine coverage in the population would stop cholera transmission, which is consistent with an earlier modeling study [16]. However, a continuous vaccination schedule might be difficult to implement, as it requires a constant effort to keep a substantial proportion of the population protected by vaccine by identifying unvaccinated individuals and vaccinating them and revaccinating individuals as protection from vaccines wane. The continuous vaccination strategy as described is a mathematical idealization of vaccination efforts that occur throughout the year rather than vaccination campaigns that occur every few years. Vaccination campaigns that occur only once a year would maintain approximately the same level of vaccine protection in the population while being more logistically practical. We also model mass vaccination campaigns that occur once every five years, the average duration of protection from vaccination [5]. This strategy might be easier to implement, but as vaccine protection wanes and birth (and possibly immigration) introduces new susceptible individuals to the population between campaign years, large cholera outbreaks can occur.

We found that vaccinating all vaccine-eligible children, ages 1–14 years, requires the fewest number of vaccinations per case averted compared to vaccinating preschool-aged children (1–4 years) or the general population (ages 1 year and older). Although preschool-aged children have the greatest burden of cholera, represented by both disease incidence and mortality [24], [35], selectively vaccinating this group is the least efficient strategy, primarily due to the lower modeled vaccine efficacy in this age group (40%) [5].

Delivering OCV to children could build upon existing delivery mechanisms like the Expanded Programme on Immunization (EPI) or National Immunization Days [3]. However, our analysis suggests that endemic cholera is unlikely to be eliminated by vaccinating only children. A major consideration of immunization plans could be the most efficient use of the limited supply of doses, currently around two million globally but anticipated to expand over the next five years [8], [9]. Vaccinating populations with the highest risk of disease is efficient and also supported by evidence from cost-effectiveness analyses, the priorities of decision makers, and health equity considerations [3], [15], [19], [36].

Previous cost-effectiveness studies have found that untargeted mass cholera vaccination in Bangladesh may not be effective unless one accounts for indirect protection [3], [13]. However, the magnitude of indirect protection is difficult to estimate without proper studies and/or mathematical modeling. The results from this study suggest significant indirect protection from OCV that may improve the economic case for expanding its use. Although we do not evaluate the cost-effectiveness of the modeled vaccination strategies, the number of vaccinations per case averted can be used to estimate cost-effectiveness. If it requires between 10–25 vaccinations per case averted (Figures 4B, 4D) and costs $5.33 to fully vaccinate an individual (two doses at a public sector cost of $1.85 per dose [3] and a delivery cost of $1.63 per individual [4]), the vaccination programs considered here would cost between $53–$133 per cholera case averted, and assuming a 1.5% case fatality rate [3], $3,500–$8,900 per death averted.

There are several limitations to this study. The model was calibrated to match the demographic and epidemiologic characteristics of cholera in Matlab, Bangladesh; so extrapolating the results from this study to other settings requires careful consideration. We modeled transmission of cholera in an endemic setting where the incidence is much higher in children than adults. However, data from cholera outbreaks in non-endemic settings suggest a more even distribution of cholera incidence by age [24], [28], [37]. Therefore, the results described apply to regions that experience annual cholera outbreaks at a scale similar to Matlab's, but the model should be recalibrated for settings with substantially different epidemiology or demography. The actual cause of the relatively high cholera incidence among children in Bangladesh is not known, but it has been hypothesized to be due to higher levels of previous exposure in adults and differences in the immune system in children and adults [38]–[40]. The model assumptions required to create this differential susceptibility could affect the effectiveness of mass vaccination. We assumed that the differences in cholera incidence between age groups were due largely to differential intrinsic susceptibility. We had tested an alternative hypothesis that the duration of immunity conferred by infection differed between age groups and could explain the differences in incidence, but this model also required differential susceptibility to fit the data from Matlab.

The model was not intended for the prediction of cholera activity for a particular year. We calibrated the model using five years of data from Matlab, assuming that the epidemiology of cholera will not change substantially, so the 20-year projections described here should be considered average outcomes over this time horizon. We assumed that current demographic trends, sanitation levels, and climate would remain constant over the next 20 years, but changes in population movement, development, rainfall, the frequency of severe flooding events, sea level, and ocean temperature could change the epidemiology of cholera [41]–[44].

There is growing momentum toward incorporating oral cholera vaccine into cholera control and outbreak response planning. Field and feasibility trials have been conducted in urban and rural Bangladesh and there appears to be interest to include targeted OCV use as part of comprehensive cholera control strategies [3], [4], [33], [45], [46]. As Bangladesh and other countries begin to consider the role of OCV in comprehensive cholera control plans, this work provides insight into how OCV may diminish cholera transmission dynamics. This analysis demonstrates that mass immunization with oral cholera vaccines may greatly reduce the burden of disease, and mathematical modeling can provide guidance on the targeting of populations and scheduling of campaigns to maximize impact.

## Supporting Information

Text S1 1. Model Description. 2. Forces of Infection. 3. Modeling Vaccination Strategies
(DOCX)

Text S2 1. **Table S1.** Parameters and ranges used in the main analysis. 2. **Table S2.** Transmission rates and relative susceptibility by age used in the model. 3. **Table S3.** Magnitude and duration of the periods of elevated environmental risk. 4. **Figure S1.** Direct vs. indirect transmission. 5. **Figure S2.** Projected variation in the proportion of individuals recovered from cholera by age group assuming no vaccination. 6. **Figure S3.** Overall effectiveness of mass vaccinations over 1 year for vaccine with 5 year of average protection assuming that: 7. **Figure S4.** Modeling 5-year campaign and continuous vaccination strategies for vaccine with 5 years of average protection assuming uniform efficacy (65%) for all age groups. 8. **Figure S5.** Modeling 3-year campaign and continuous vaccination strategies for vaccine with 3 years of average protection assuming uniform efficacy (65%) for all age groups. 9. **Figure S6.** Model dynamics assuming 25% of cholera cases are reported. 10. **Figure S7.** The effectiveness of different vaccination strategies for vaccine with 5 years of average protection assuming that 25% of cholera cases are reported. 11. **Figure S8.** Model dynamics assuming the duration of protection after cholera infection varies across age groups. 12. **Figure S9.** Effectiveness of different vaccination strategies for vaccine with 5 years of average protection assuming different duration of natural immunity across age groups.
(DOCX)
