# Supplementary material for: Comparative Effectiveness of Different Strategies of Oral Cholera Vaccination in Bangladesh: A Modeling Study
Source: PLoS Negl Trop Dis. 2014 Dec 4;8(12):e3343. doi: 10.1371/journal.pntd.0003343 (PMC4256212; doi:10.1371/journal.pntd.0003343)
Supplement: Text S1 — 1. Model Description. 2. Forces of Infection. 3. Modeling Vaccination Strategies (DOCX) [file pntd.0003343.s001.docx]

**Text S1.**

**1. Model description**

**The model is described by a set of differential equations for each age group (i=1 for infants age 0-1 years, i=2 for preschoolers age 2-4 years, i=3 for school children 5-14 years, and i=4 for adults age 15+ years):**

$$\begin{matrix} \frac{dS_{i}}{dt}=b_{i}N+a_{i-1}S_{i-1}+\gamma_{3}R_{i}+\nu_{2}V_{i}-(\lambda_{i}+\nu_{1}+\mu_{i}+a_{i})S_{i} \\ \frac{dV_{i}}{dt}=\nu_{1}(S_{i}+R_{i})+a_{i-1}V_{i-1}-(\lambda{}_{i}^{V}+\nu_{2}+\mu_{i}+a_{i}) V_{i} \\ \frac{dI_{i}}{dt}=p\lambda_{i}S_{i}+q\lambda{}_{i}^{V}V_{i}+a_{i-1}I_{i-1}-(\mu_{i}+d+\gamma_{1} +a_{i})I_{i} \\ \frac{dA_{i}}{dt}=(1-p)\lambda_{i}S_{i}+(1-q)\lambda{}_{i}^{V}V_{i}+a_{i-1}A_{i-1}-(\mu_{i}+\gamma_{2} +a_{i})A_{i} \\ \frac{dR_{i}}{dt}=\gamma_{1}I_{i}+\gamma_{2}A_{i}+a_{i-1}R_{i-1}-(\mu_{i}+\nu_{1}+\gamma_{3}+a_{i}) R_{i} \\ \frac{dW}{dt}=\xi_{1}\sum_{i=1}^{4} I_{i}+\xi_{2}\sum_{i=1}^{4} A_{i}-\theta W \end{matrix}$$

b_i_- birth rate, contributes to the infant group only (b_1_>0, b_i_=0 for i=2,3,4)

*a_i_* – maturation rates from group i to group i+1

ν_1_- vaccination rate

ν_2_- loss of vaccine immunity

γ*_1,_* γ*_2_*- recovery rates for cholera cases and asymptomatic infections

γ*_3_*- loss of natural immunity

*ξ_1_, ξ_2_* - excretion of *V. cholerae* by cholera cases and asymptomatically infected in water

*θ*- decay rate of *V. cholerae* in the environment

μ_i_- natural death rate by age group

*p* – proportion of the infections of non-vaccinated which become cholera cases

*q* – proportion of the infections of vaccinated which become symptomatically infected

N – total population size

**2. Forces of infection**

The forces of infections ($\lambda_{i} and \lambda_{i}^{V}$) represent the risk of the susceptible and vaccinated individuals in age group i to acquire cholera including short cycle transmission through contacts with infected individuals and long cycle transmission through exposure to contaminated water:

$$\begin{matrix} \lambda_{i}=(\sum_{j=1}^{4} I_{j}+\beta_{asymp}\sum_{j=1}^{4} A_{j})\frac{s_{i}\beta_{H}}{N}+\frac{s_{i}\beta_{W}W}{k+W} \\ \lambda_{i}^{V}=(\sum_{j=1}^{4} I_{j}+\beta_{asymp}\sum_{j=1}^{4} A_{j})\frac{(1-VE_{S})s_{i}\beta_{H}}{N}+\frac{(1-VE_{S})s_{i}\beta_{W}W}{k+W} \\ \beta_{W}=\left\{\begin{matrix} \beta_{W}^{\min}(1+\delta_{1}) rem(t,360)<60 \\ \beta_{W}^{\min}(1+\delta_{2}) \delta_{3}+150<rem(t,360)<\delta_{3}+210 \\ b_{W}^{\min} otherwise \end{matrix}\right. \end{matrix}$$

where

*β_H_* – transmission rate from cholera cases to unvaccinated susceptible adults by direct contact (human-to-human)

β_asymp_ – relative infectiousness of the asymptomatically infected.

$\beta_{W}^{min}$ - transmission rate to susceptibles adults due to the environment (water-to-human) outside of cholera season

δ_1_ (δ_2_) – seasonal multiplier for increased environmental risk during spring (fall) seasons

δ_3_ – shift in the start of the season of elevated environmental risk (fall)

*β_W_* – transmission rate to susceptible adults from the environment (water-to-human)

s_i_ (i=1..4) – relative susceptibility of the group i. Adult group is used as a baseline (s_4_=1)

VE_S_ – vaccine efficacy in reducing susceptibility

**3. Modeling vaccination strategies**

All simulations are initiated with the population distributed among compartments based on the procedure described below. Simulations without vaccination are initiated with (V=0) and no transfers to the vaccinated compartment (ν_1_=0). The following vaccination strategies have been simulated with the model:

**One time vaccination campaign**. A proportion (coverage) of the targeted population is vaccinated once at the start of the simulation. Modeled by direct transfer of proportion k (coverage) of S and R compartments into V at the start (V(0)=kS(0)+kR(0)). The number of vaccinations is given by V(0).

**Periodic campaigns.** A proportion of the targeted population is vaccinated every three years. Modeled by direct transfer of proportion k (coverage) of S and R compartments into V at regular 5- or 3-year intervals. The individuals still remaining in the vaccinated compartment (V) immediately before the next campaign are also eligible for revaccination. The population distribution after each vaccination campaign (S_new_, R_new_, V_new_) is related to the population distribution before the campaign (S_old_, R_old_, V_old_) as follows (V_new_=V_old_+kS_old_+kR_old_, S_new_=(1-k)S_old_, R_new_=(1-k)R_old_). The number of vaccinations per campaign is given by kV_old_+kS_old_+kR_old_

**Continuous vaccination.** A proportion k of the targeted population is vaccinated initially (V(0)=kS(0)+kR(0)) and after the first year, a proportion of S and R are vaccinated at a fixed rate. Vaccination rate ν_1_ is set (ν_1_=0) for the first year and replaced with (ν_1_= $\frac{k}{3-2k}\mathrm{or}\nu_{1}= \frac{k}{5-4k}$) afterward, where the value of ν_1_ is selected to balance the proportion of vaccinated who lose protection in one year if the vaccine is protective for an average of 3 and 5 years respectively. The total number of vaccinations over T years is given by ${\mathrm{kS}\left( 0 \right)+kR\left( 0 \right)+\int_{1}^{T} \nu}_{1}\left( S\left( t \right)+R\left( t \right) \right)dt$.

The vaccination of one-year-olds in all scenarios is modeled by vaccinating half of the cohort younger than 2 years.
