# Supplementary material for: Comparative Effectiveness of Different Strategies of Oral Cholera Vaccination in Bangladesh: A Modeling Study
Source: PLoS Negl Trop Dis. 2014 Dec 4;8(12):e3343. doi: 10.1371/journal.pntd.0003343 (PMC4256212; doi:10.1371/journal.pntd.0003343)
Supplement: Text S2 — 1. Table S1. Parameters and ranges used in the main analysis. 2. Table S2. Transmission rates and relative susceptibility by age used in the model. 3. Table S3. Magnitude and duration of the periods of elevated environmental risk. 4. Figure S1. Direct vs. indirect transmission. 5. Figure S2. Projected variation in the proportion of individuals recovered from cholera by age group assuming no vaccination. 6. Figure S3. Overall effectiveness of mass vaccinations over 1 year for vaccine with 5 year of average protection assuming that: 7. Figure S4. Modeling 5-year campaign and continuous vaccination strategies for vaccine with 5 years of average protection assuming uniform efficacy (65%) for all age groups. 8. Figure S5. Modeling 3-year campaign and continuous vaccination strategies for vaccine with 3 years of average protection assuming uniform efficacy (65%) for all age groups. 9. Figure S6. Model dynamics assuming 25% of cholera cases are reported. 10. Figure S7. The effectiveness of different vaccination strategies for vaccine with 5 years of average protection assuming that 25% of cholera cases are reported. 11. Figure S8. Model dynamics assuming the duration of protection after cholera infection varies across age groups. 12. Figure S9. Effectiveness of different vaccination strategies for vaccine with 5 years of average protection assuming different duration of natural immunity across age groups. (DOCX) [file pntd.0003343.s002.docx]

**Text S2.**

**Table S1. Parameters and ranges used in the main analysis.**

| **Parameter** | **Description** | **Values and ranges** | **Ref.** |
| --- | --- | --- | --- |
| ***γ_1_ =γ_2_*** | Recovery rates (daily) for cholera cases and asymptomatic infections (infectious time)^-1^ | (5 days)^-1^ | [[1](#_ENREF_1)] |
| ***γ_3_*** | Loss (daily) of natural immunity (protected time)^-1^ | (3 years)^-1^ | [[2](#_ENREF_2)] |
| b_i_ | Birth rate (annual) | 0.025 | [[3](#_ENREF_3)] |
| ***N*** | Initial population size | 220,000 | [[3](#_ENREF_3)] |
| μ_i_ | Death rates (annual) by age groups (0-1,2-4,5-14,15+) | 2%, 0.2%, 0.1% 1% | [[3](#_ENREF_3)] |
| **k** | Half-saturation concentration of cholera in the environment (water) | 10^5^ | [[4](#_ENREF_4)] |
| ***ξ_1_, ξ_2_*** | Excretion rate (daily) of cholera by cholera cases [asymptomatically infected] in water | 100 [1] | [[5](#_ENREF_5),[6](#_ENREF_6)] |
| ***θ*** | Death rate (daily) of Cholera in water (survival time)^-1^ | (30 days)^-1^ | [[4](#_ENREF_4)] |
| **p** | Proportion of the infections of non-vaccinated which become cholera cases | 20% | Assumption |
| **q** | Proportion of the infections of vaccinated which become cholera cases | 20% | Assumption |
| ***ν_2_*** | Loss (daily) of vaccine immunity (protected time)^-1^ | (5 years)^-1^ | [[7](#_ENREF_7)] |
| **VE_S_** | Vaccine efficacy in reducing susceptibility | 65% (5+ years)  40% (1-4 years) | [[7](#_ENREF_7),[8](#_ENREF_8)] |
| **r** | Proportion cholera cases reported | 10% | Assumption |
| **InitR** | Fraction initially recovered | 18.1% | [[9](#_ENREF_9)] |

**References**

1. Sack DA, Sack RB, Nair GB, Siddique AK (2004) Cholera. The Lancet 363: 223-233.

2. Ali M, Emch M, Park JK, Yunus M, Clemens J (2011) Natural cholera infection-derived immunity in an endemic setting. J Infect Dis 204: 912-918.

3. Rahman M, Alam N, Razzaque A, Streatfield PK (2012) Health and Demographic Surveillance System- Matlab. Mohakhali, Dhaka, Bangladesh: International Center for Diarrheal Disease Research, Bangladesh.

4. Hartley DM, Morris JG, Jr., Smith DL (2006) Hyperinfectivity: a critical element in the ability of V. cholerae to cause epidemics? PLoS Med 3: e7.

5. Andrews JR, Basu S (2011) Transmission dynamics and control of cholera in Haiti: an epidemic model. The Lancet 377: 1248-1255.

6. Nelson EJ, Harris JB, Morris JG, Jr., Calderwood SB, Camilli A (2009) Cholera transmission: the host, pathogen and bacteriophage dynamic. Nat Rev Microbiol 7: 693-702.

7. Bhattacharya SK, Sur D, Ali M, Kanungo S, You YA, et al. (2013) 5 year efficacy of a bivalent killed whole-cell oral cholera vaccine in Kolkata, India: a cluster-randomised, double-blind, placebo-controlled trial. The Lancet Infectious Diseases 13: 1050-1056.

8. Sinclair D, Abba K, Zaman K, Qadri F, Graves PM (2011) Oral vaccines for preventing cholera. Cochrane Database Syst Rev: CD008603.

9. Giebultowicz S, Ali M, Yunus M, Emch M (2011) A comparison of spatial and social clustering of cholera in Matlab, Bangladesh. Health Place 17: 490-497.

**Table S2. Transmission rates and relative susceptibility by age used in the model.**

|  | Transmission rates | | | Relative susceptibility | | |
| --- | --- | --- | --- | --- | --- | --- |
| Parameters | From cholera cases | From asymptomatic infection | From environment | Relative  suscept.  (0-1y) | Relative  suscept.  (2-4y) | Relative  suscept.  (5-14y) |
| Best fit | 0.4477 | 0.0651 | 2.258 x10^-6^ | 6.274 | 5.253 | 1.845 |

**Table S3. Magnitude and duration of the periods of elevated environmental risk**

| Year | Magnitude first period (δ_1_) | Magnitude second period (δ_2_) | Delay in second period (δ_3_) |
| --- | --- | --- | --- |
| 1997 | 39.42987 | 72.05469 | 0.298722 |
| 1998 | 56.96794 | 145.9644 | 0.062476 |
| 1999 | 65.45569 | 32.58493 | 35.37887 |
| 2000 | 7.25094 | 21.42262 | 41.36268 |
| 2001 | 11.63899 | 1.074904 | 48.53861 |
| **Range** | **10-80** | **15-150** | **0-60** |


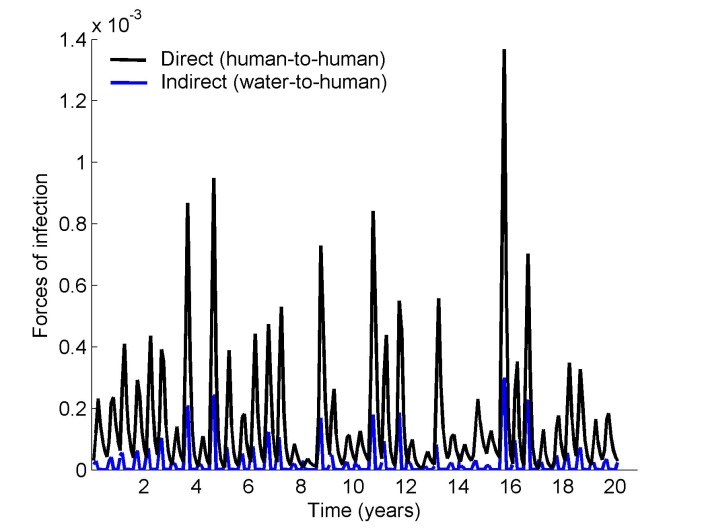

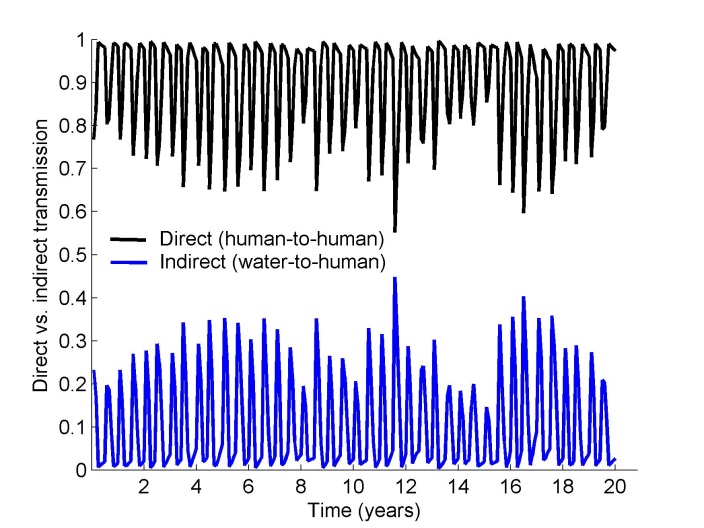


A)

B)

**Figure S1. Direct vs. indirect transmission.** A) Force of infection from short cycle (human-to-human) and long cycle (water-to-human) transmission routes over a 20-year model run. B) Relative fraction of transmissions from the two transmission routes for the same run. Water-to-human transmission is amplified twice per year using step functions, which represent monsoon cycles. Parameters of seasonal environmental exposure are sampled from ranges in Table S3.


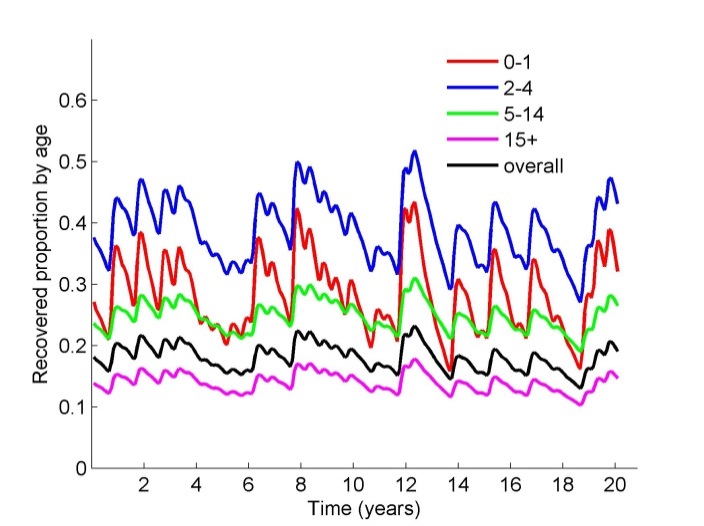


**Figure S2. Projected variation in the proportion of individuals recovered from cholera by age group assuming no vaccination**.


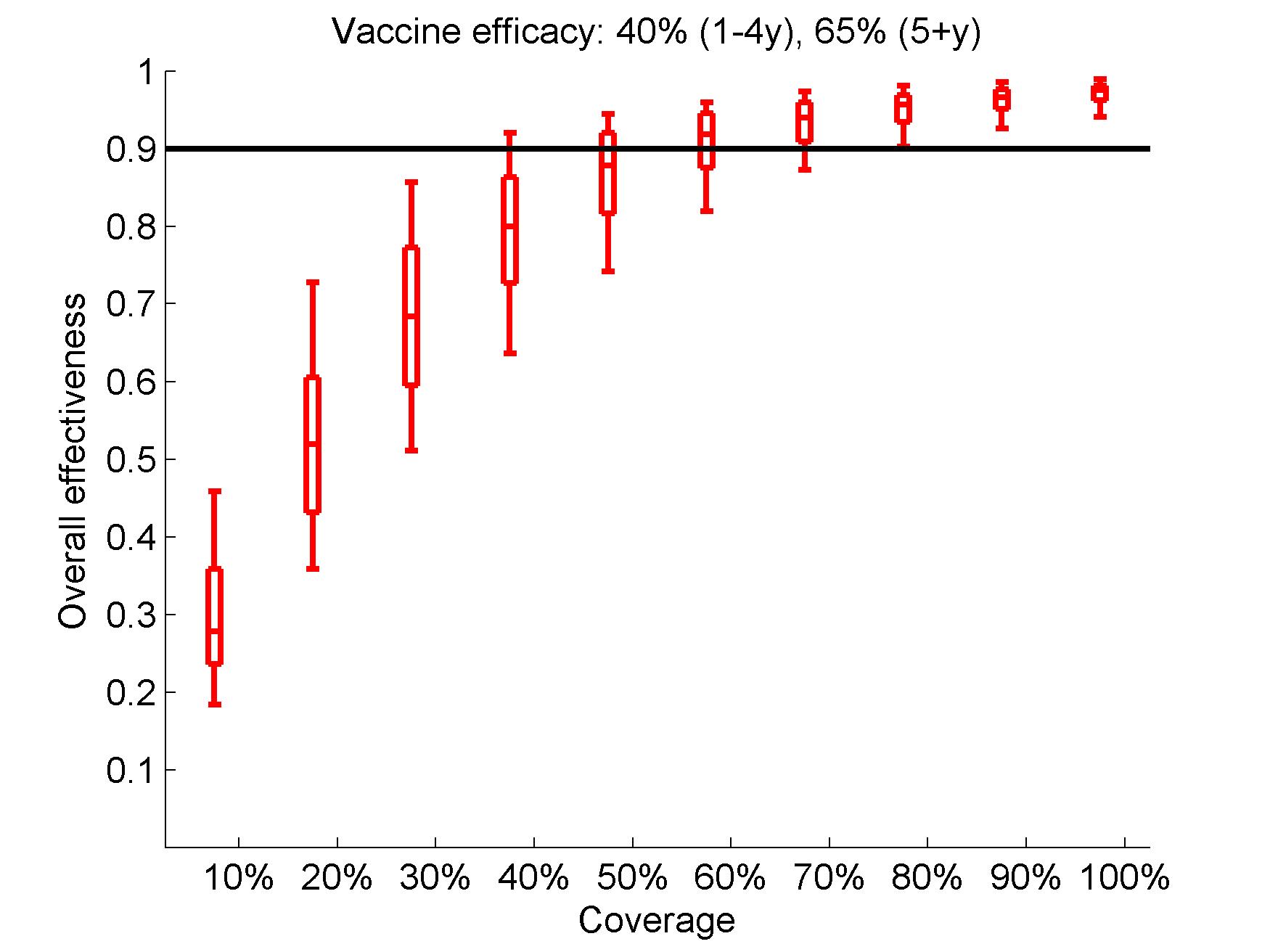

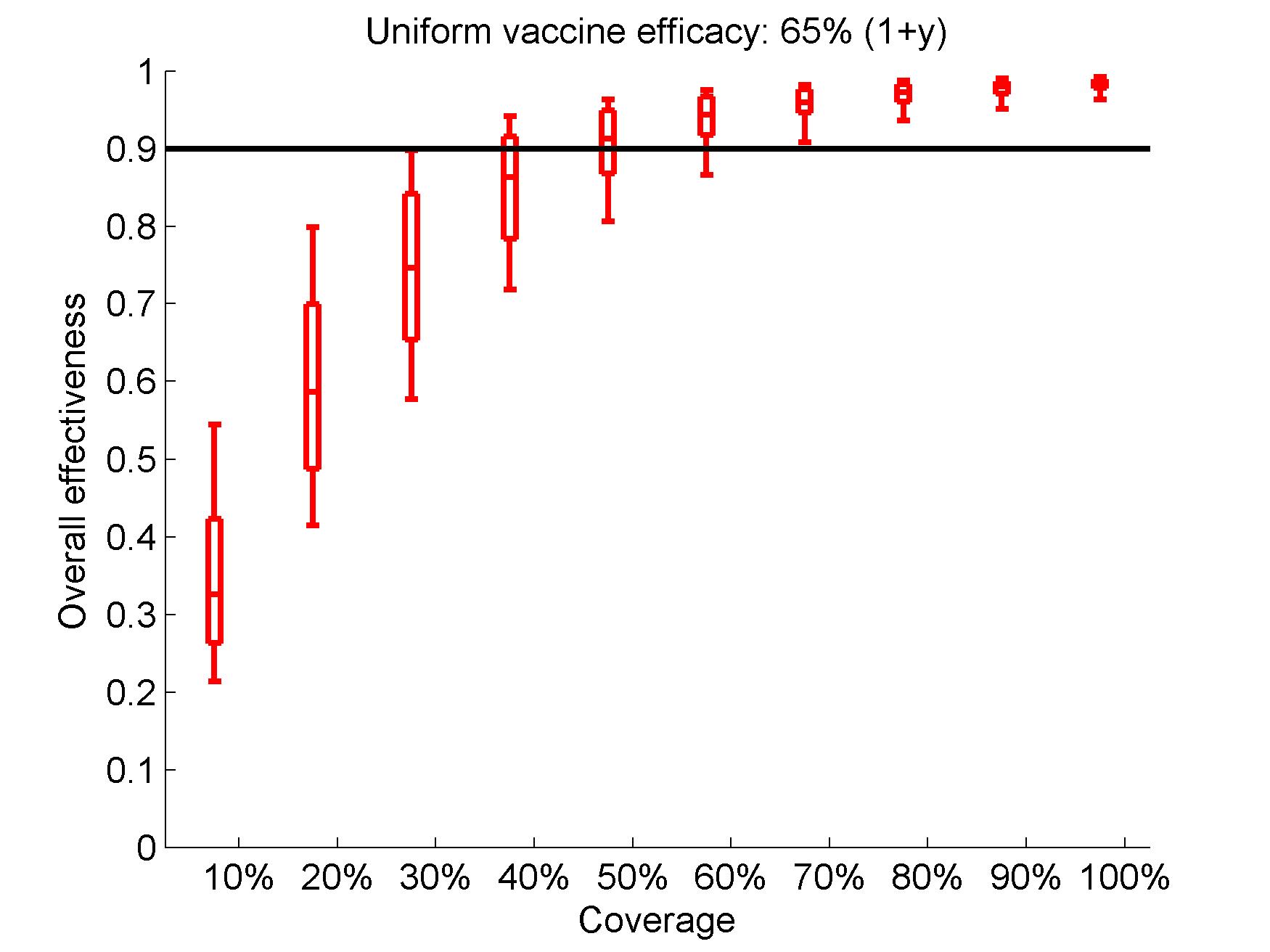


A)

B)

**Figure S3. Overall effectiveness of mass vaccinations over 1 year for vaccine with 5 year of average protection assuming that:** A) Children aged 1-4 years old have a lower vaccine efficacy (40%) than older children and adults (65%) or B) Uniform vaccine efficacy (65%) for all ages. Boxes represent the interquartile range and whiskers cover 90% of the results from 100 simulations per scenario. Note that 50% coverage is enough to eliminate roughly 90% of the cholera cases.

B)

D)


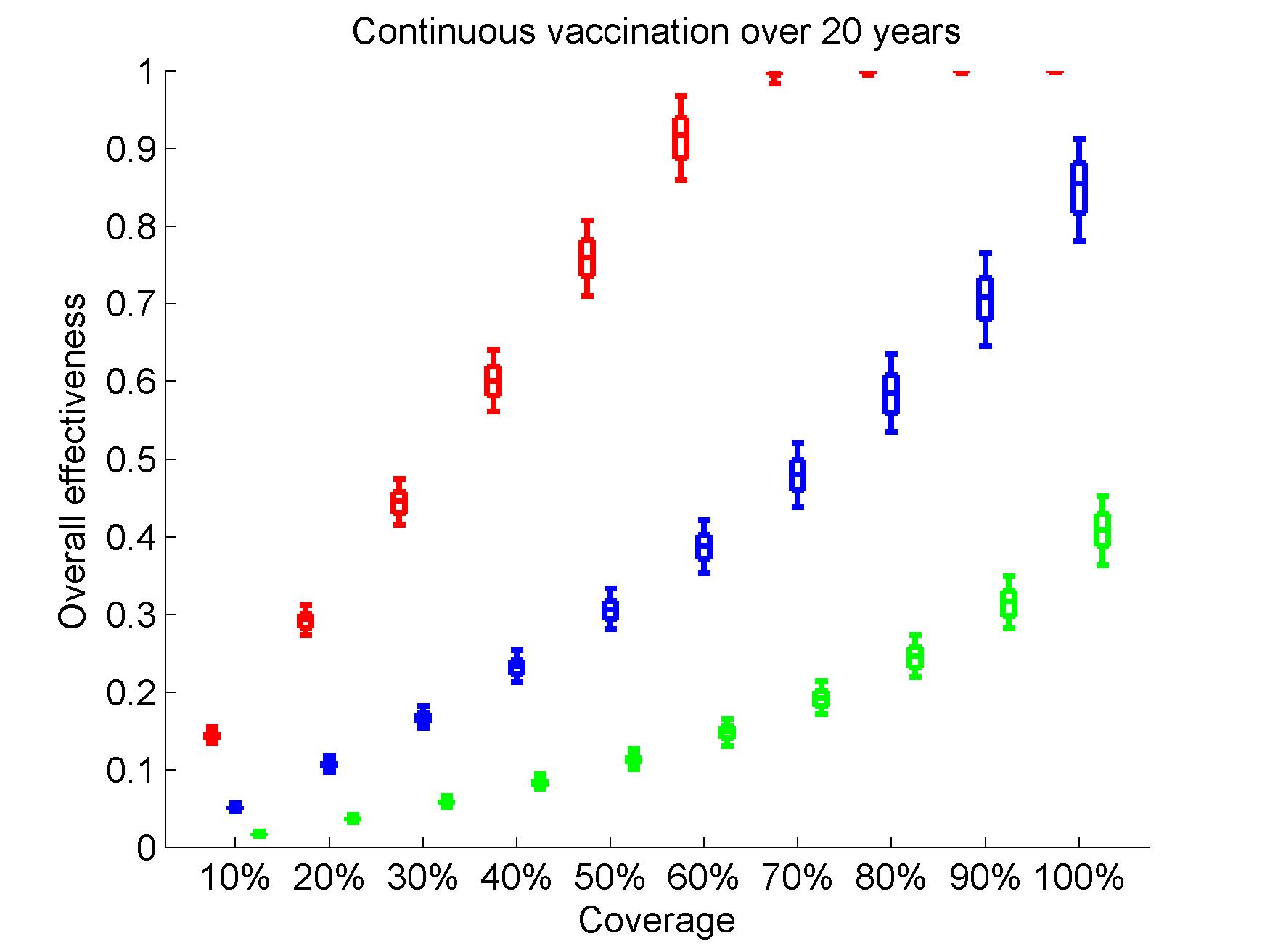

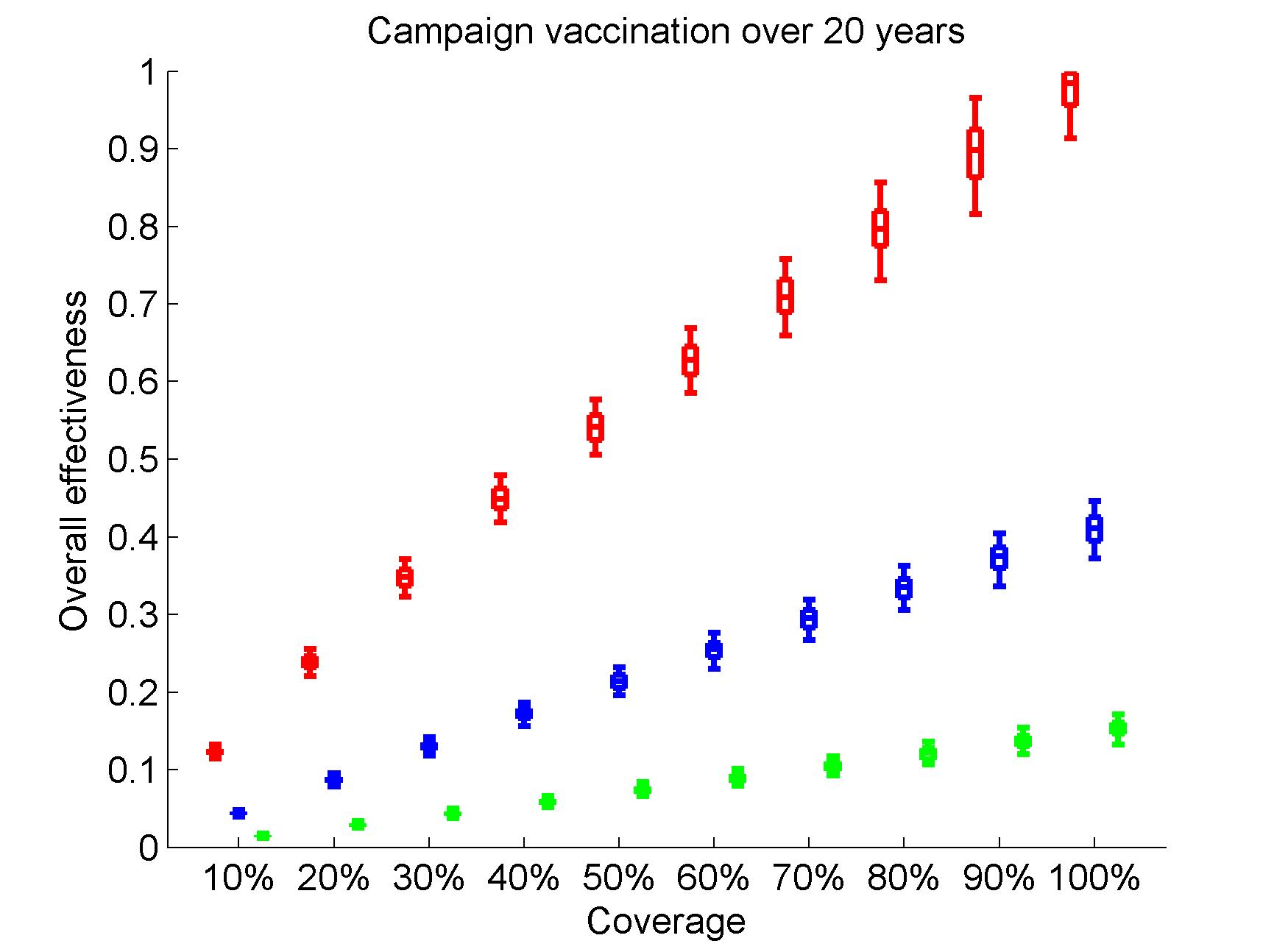


A)

C)

D)


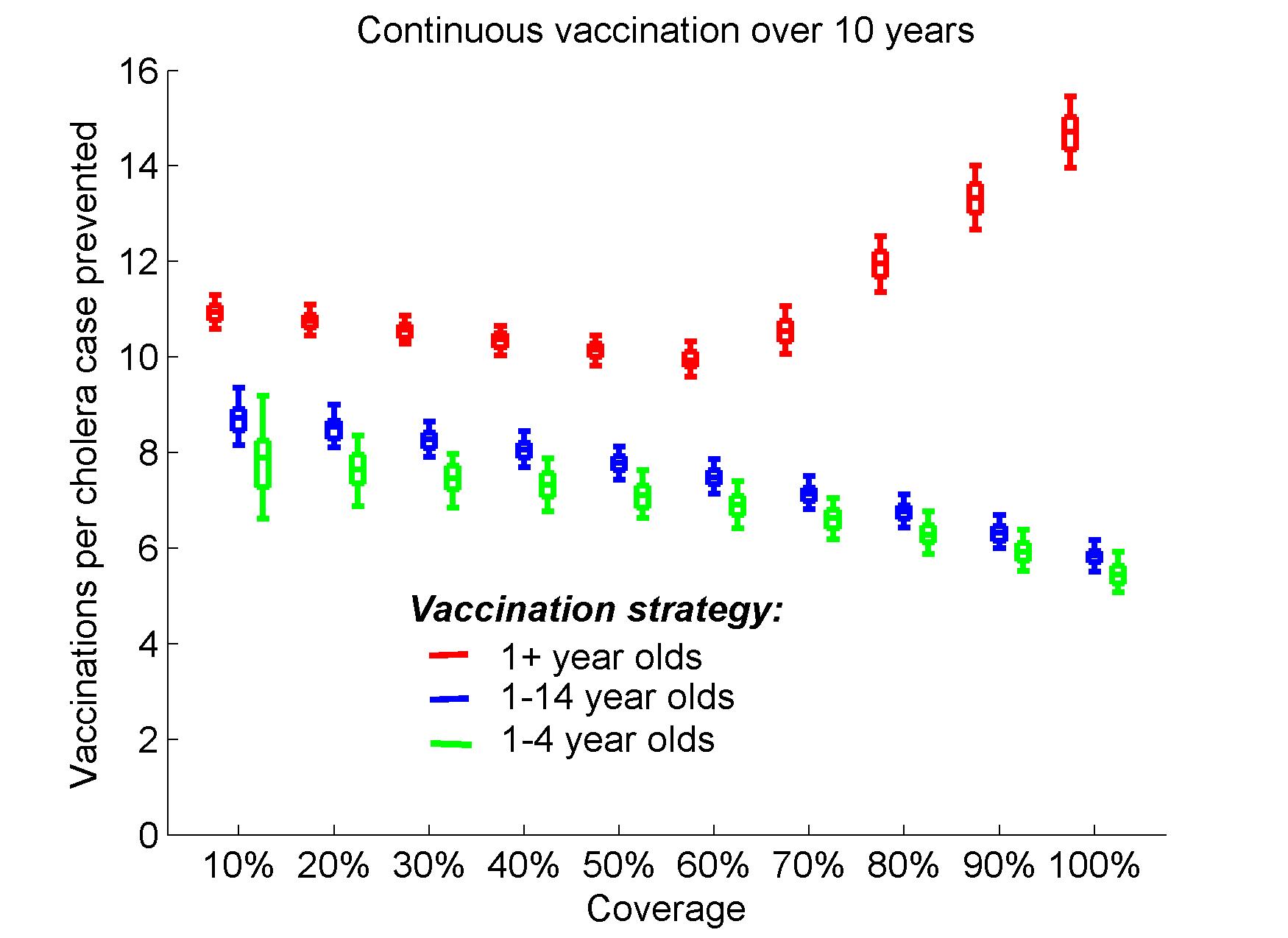

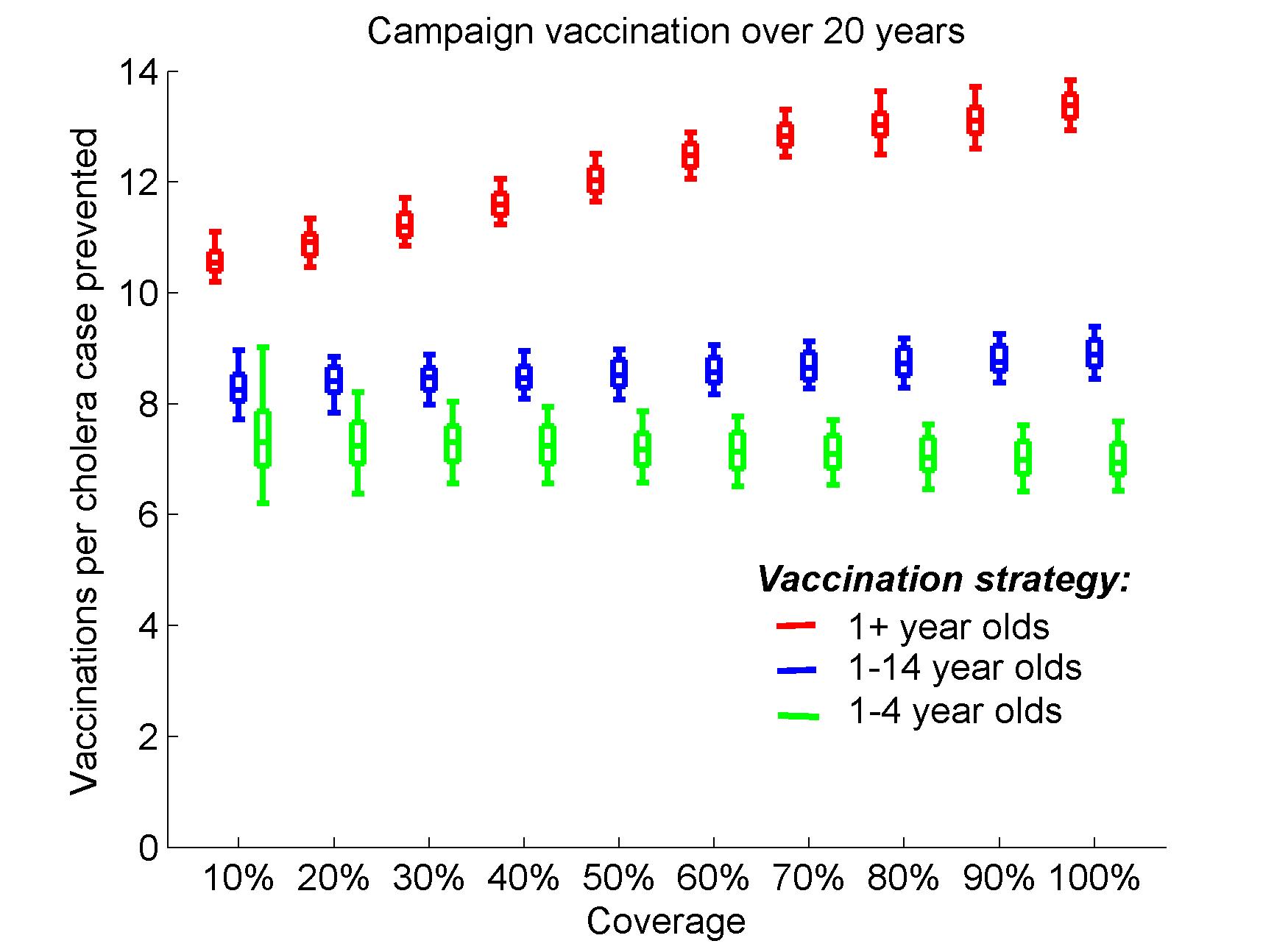


**Figure S4. Modeling 5-year campaign and continuous vaccination strategies** for vaccine with 5 years of average protection assuming uniform efficacy (65%) **for all age groups.** A) Overall effectiveness and B) vaccinations per case prevented by mass campaign vaccinations every 5 years. C) Overall effectiveness and D) vaccinations per case prevented by continuous vaccinations. Dashed lines show the effectiveness if only direct effects of the vaccine are considered. Overall effectiveness is measured in terms of prevented fraction of cholera cases over 20 years. Boxes represent the interquartile range and the whiskers cover 90% of the results from 100 simulations per scenario with parameters of seasonal environmental exposure sampled from ranges in Table S3.

D)


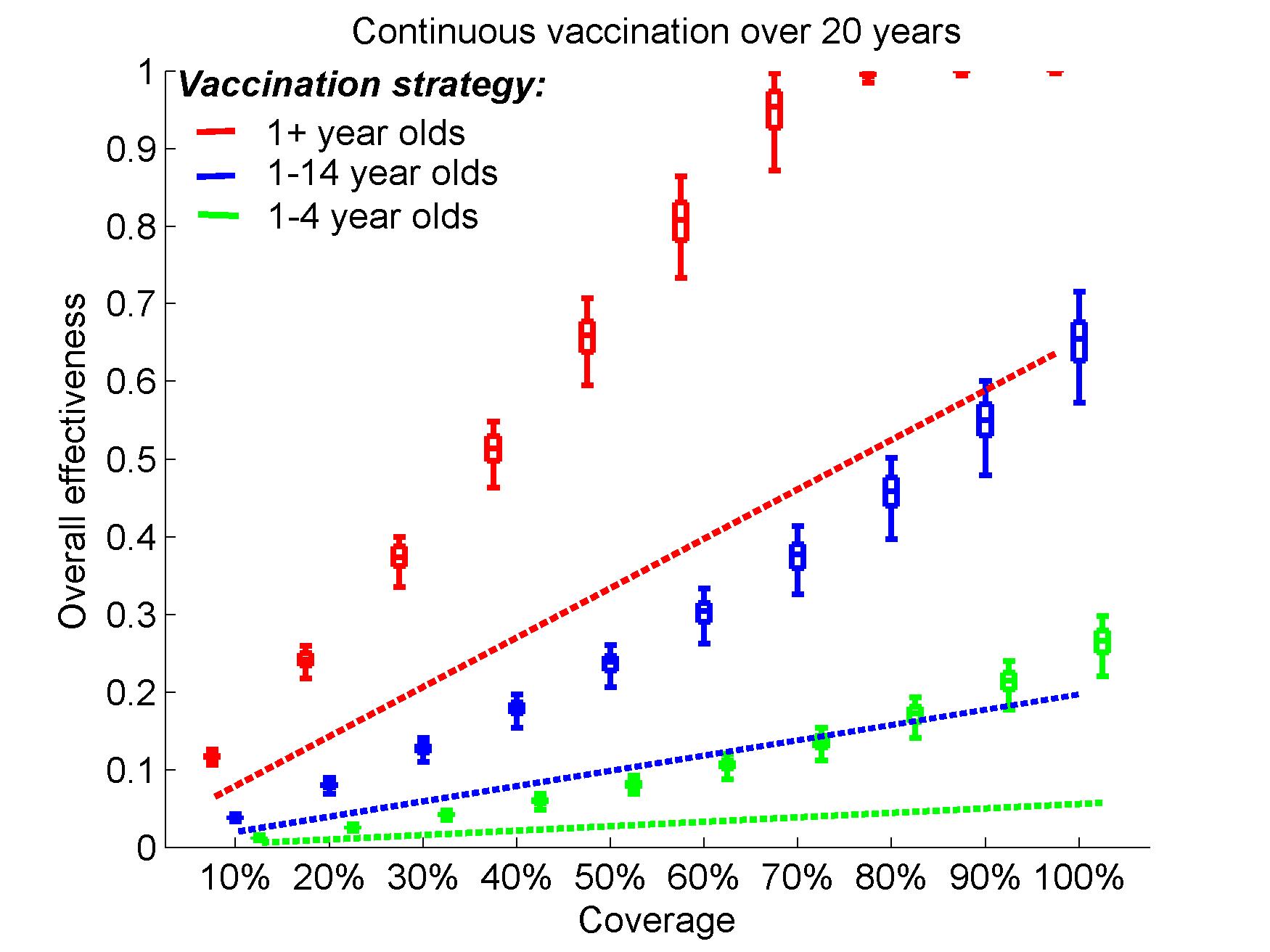

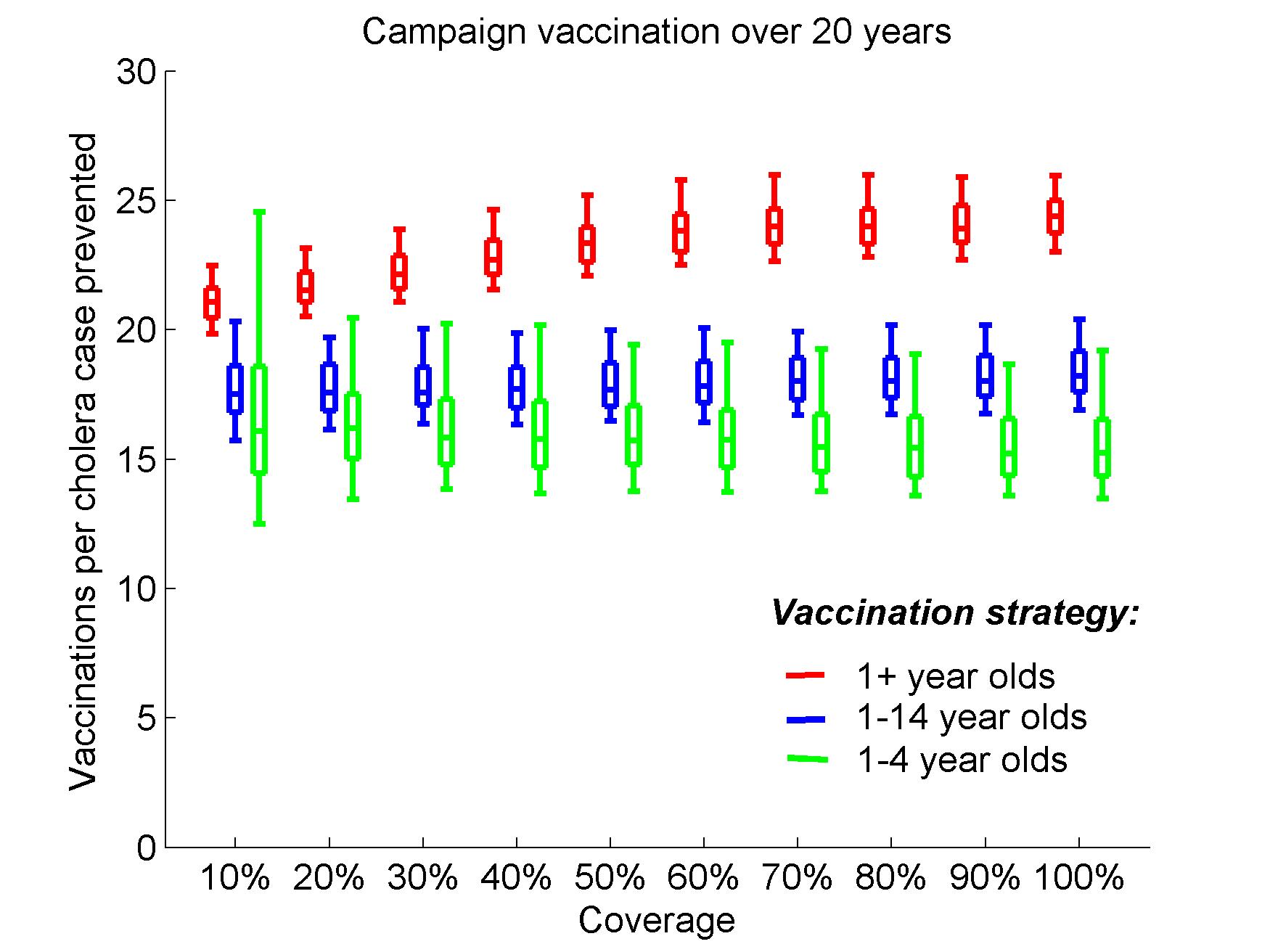

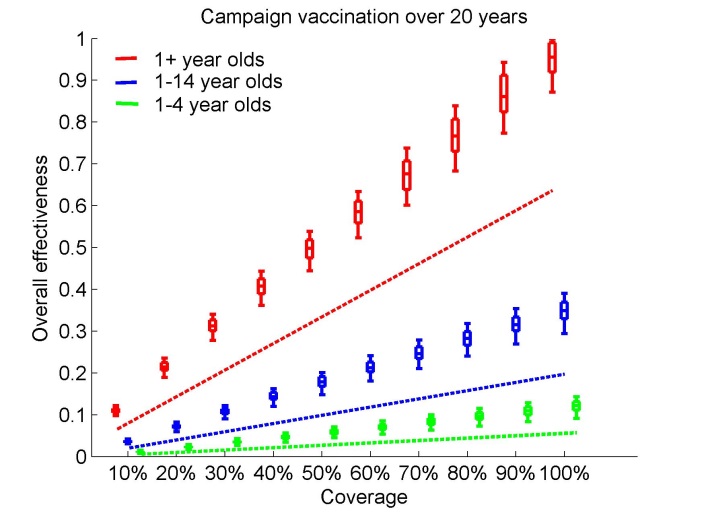


A)

B)

C)

D)


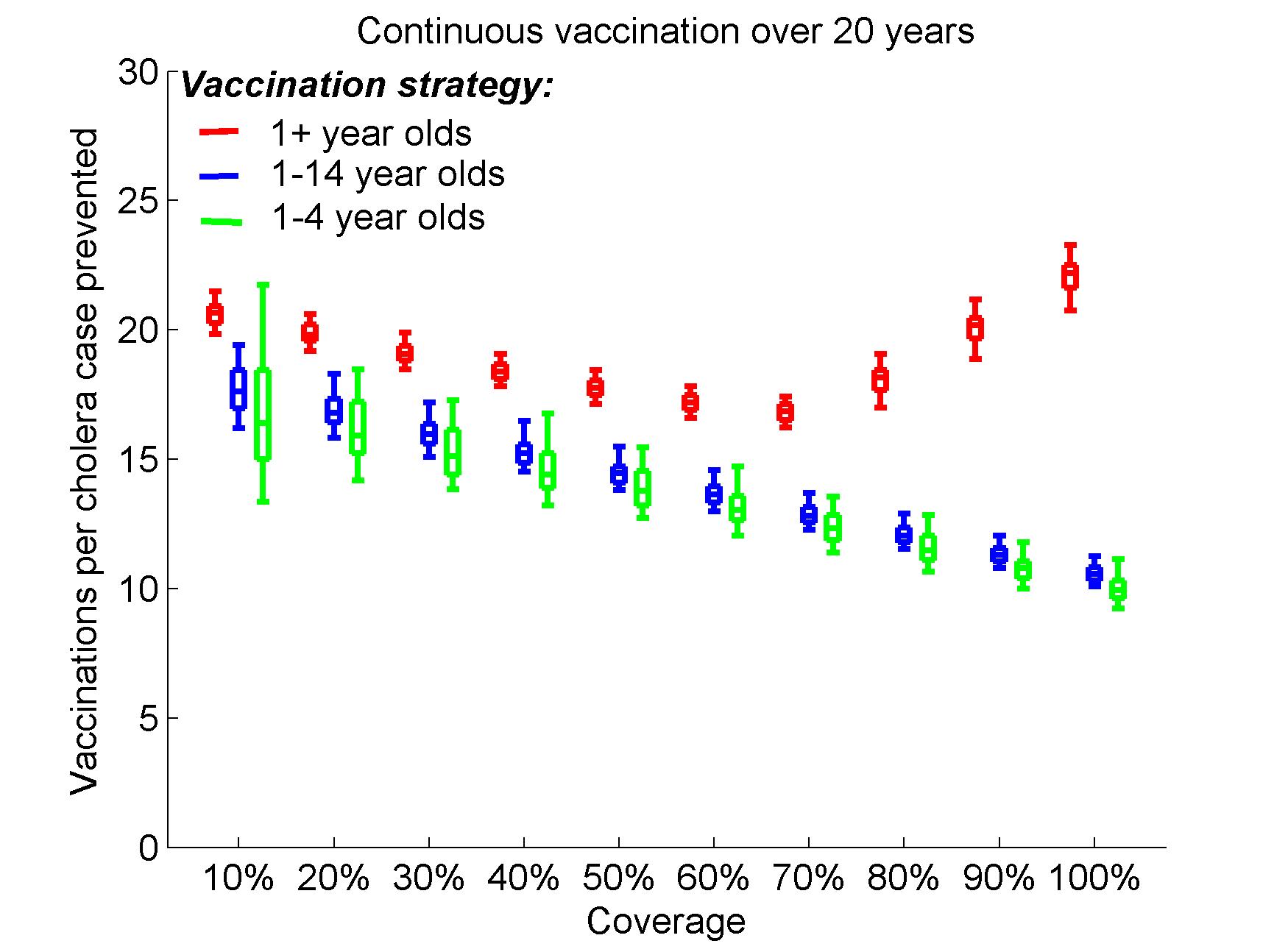


**Figure S5. Modeling 3-year campaign and continuous vaccination strategies** for vaccine with 3 years of average protection assuming uniform efficacy (65%) **for all age groups.** A) Overall effectiveness and B) vaccinations per case prevented by mass campaign vaccinations every 3 years. C) Overall effectiveness and D) vaccinations per case prevented by continuous vaccinations. Dashed lines show the effectiveness if only direct effects of the vaccine are considered. Overall effectiveness is measured in terms of prevented fraction of cholera cases over 20 years. Boxes represent the interquartile range and the whiskers cover 90% of the results from 100 simulations per scenario with parameters of seasonal environmental exposure sampled from ranges in Table S3.

.


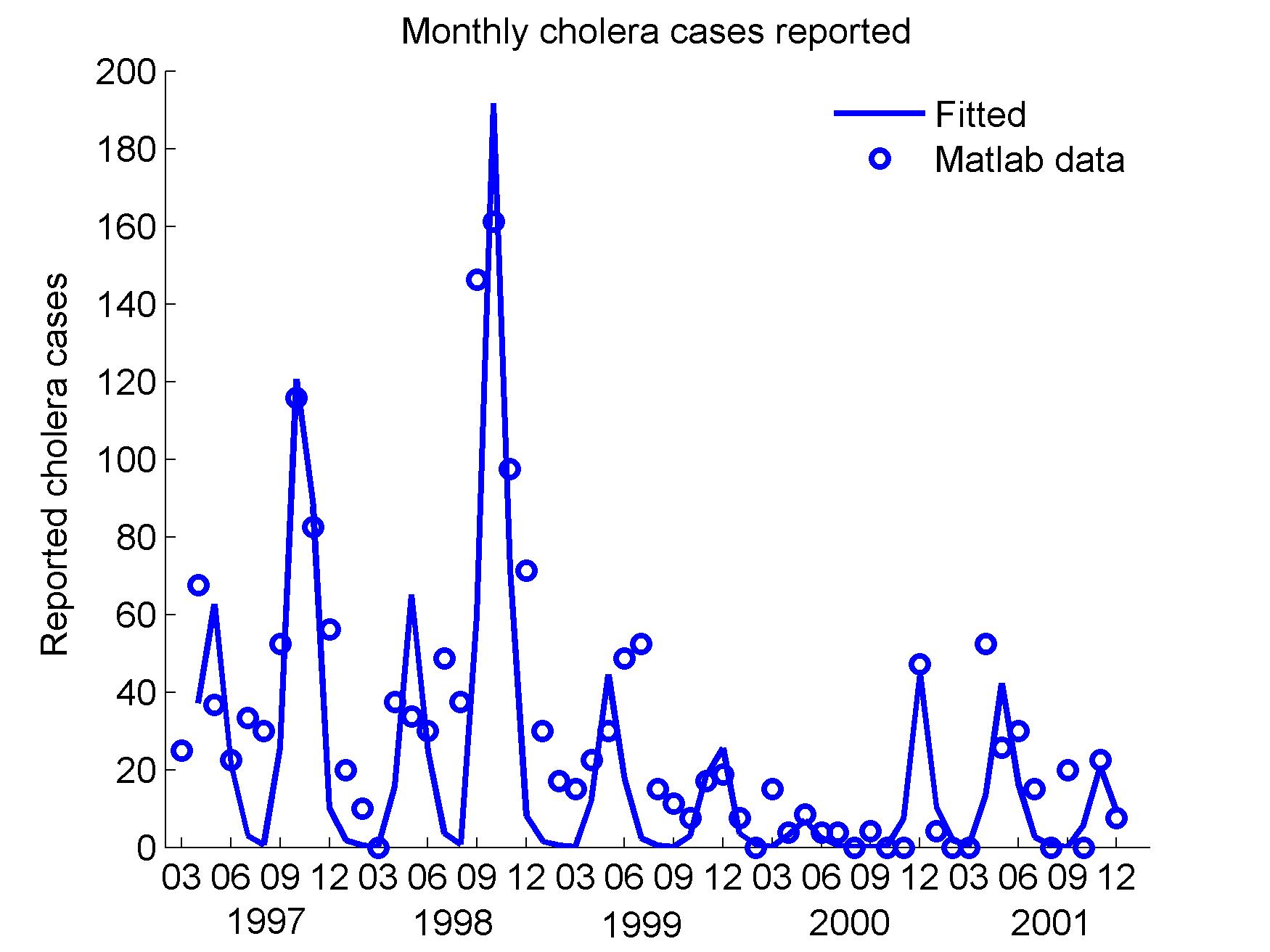


A)


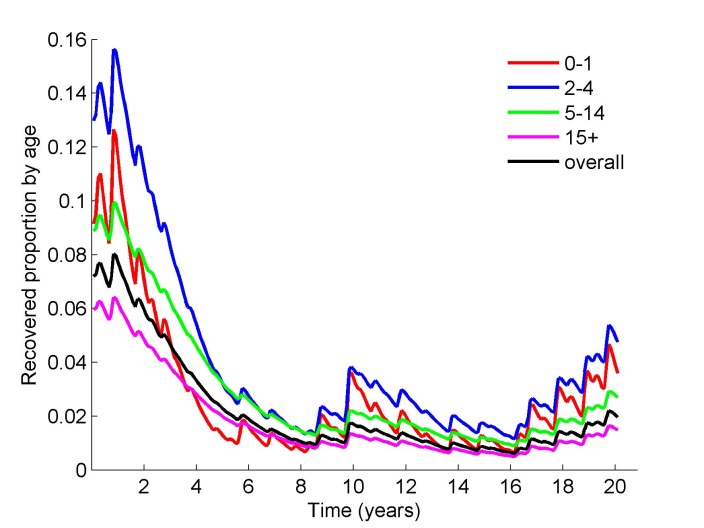


B)

**Figure S6. Model dynamics assuming 25% of cholera cases are reported.**  A) Fitting the reported cholera cases per month in Matlab from March 1997 to December 2001 assuming that 25% of the cholera cases are reported**.** B) Projected variation in the proportion of individuals recovered from exposure to cholera with temporal immunity by age group assuming no vaccination.

B)


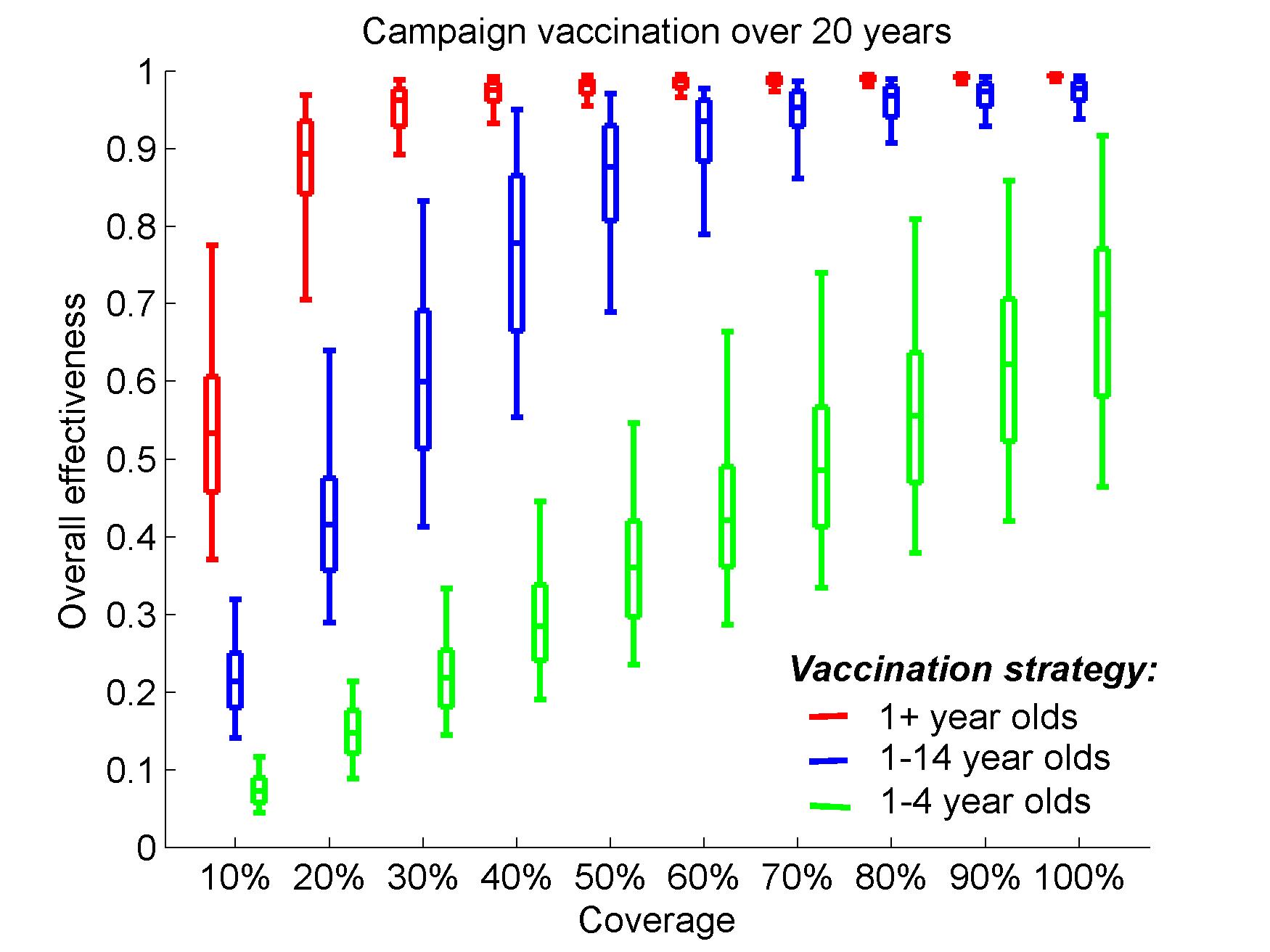

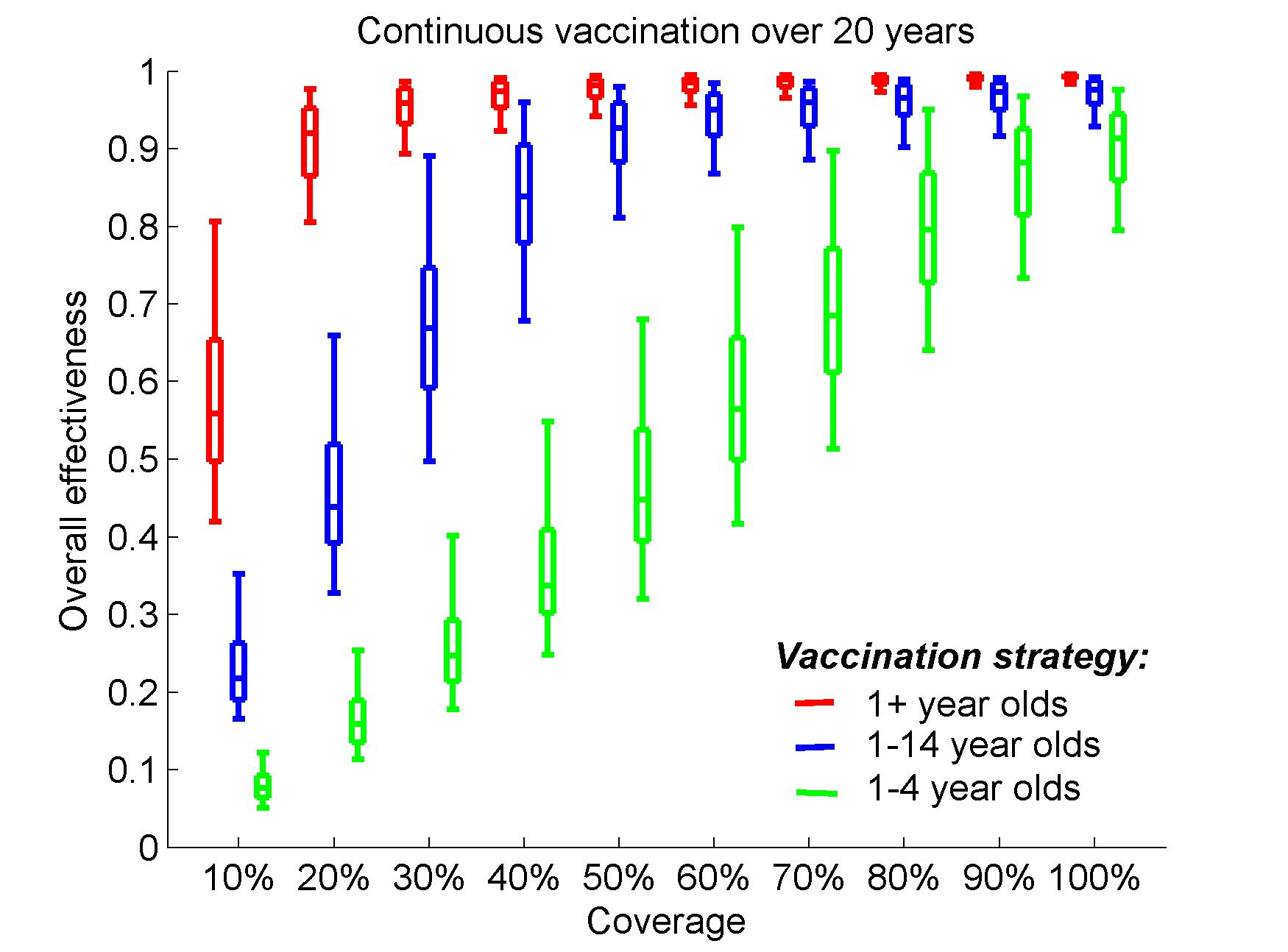


A)

**Figure S7. The effectiveness of different vaccination strategies** for vaccine with 5 years of average protection **assuming that 25% of cholera cases are reported.** Vaccine is less effective for children 1-4 years old (40% efficacy) than for older children and adults (65% efficacy). Overall effectiveness of A) 5-year campaign vaccinations and B) continuous vaccinations over 20 years. The boxes represent the interquartile range and the whiskers cover 90% of the results from 100 simulations per scenario. Note that 30% coverage of the population one year and older is enough for both, campaign and continuous, vaccination to prevent 90% of the cholera cases. Also, it suggests that campaign vaccination of 80% of the children (1-14 years old) and 60% continuously will be enough to prevent 90% of the cholera cases in the population.


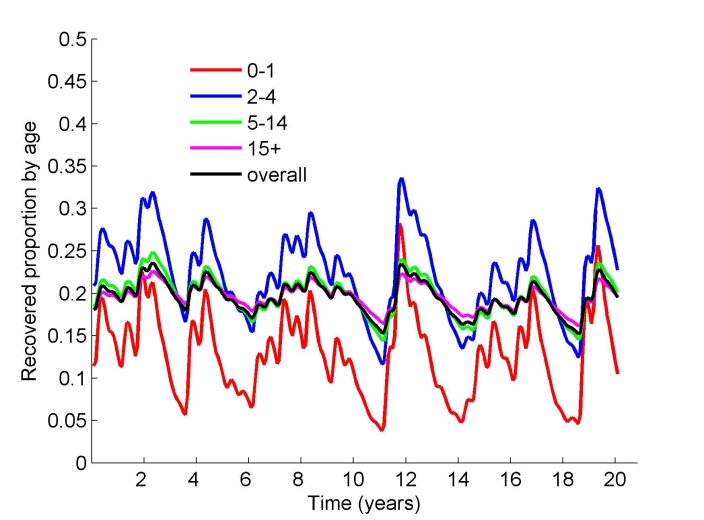

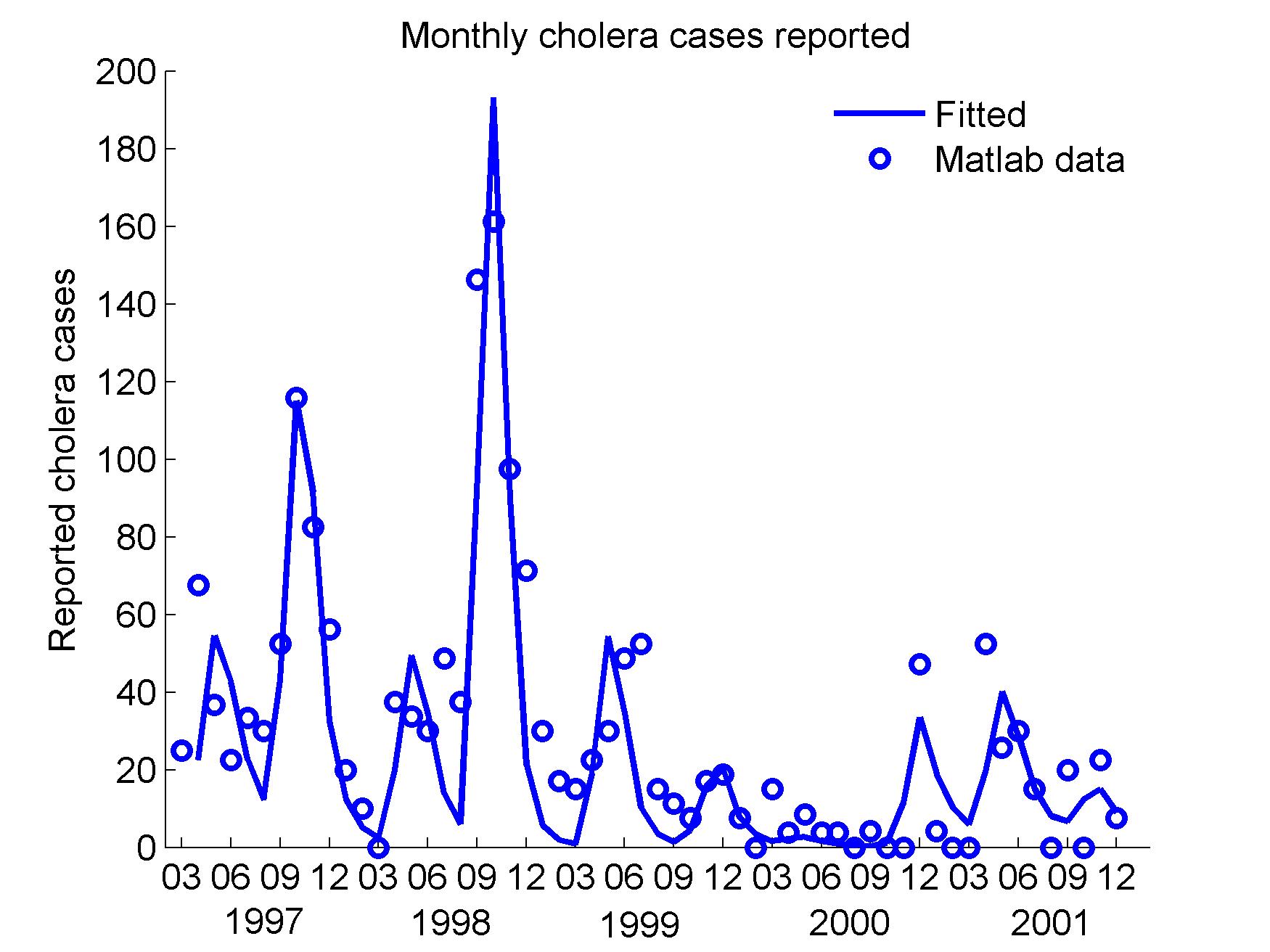


A)

B)

**Figure S8. Model dynamics assuming the duration of protection after cholera infection varies across age groups.** Here we assume 10% of the cholera cases are reported and the duration of natural immunity differs across age groups (1y, 2y, 3y, and 5y, respectively for age groups 0-1, 2-4, 5-14, and 15+ years old). A) Fitting the reported cholera cases per month in Matlab from March 1997 to December 2001. B) Projected variation in the proportion of individuals recovered from exposure to cholera with temporal immunity by age group assuming no vaccination. Note that the recovered fractions follow qualitatively different patterns compared to the main scenario assuming the same rate of waning of natural immunity across age groups. The fraction of the population that is Recovered is highest is among toddlers (blue) but much smaller fraction among infants (red) with older age groups following closely the overall fraction across population. Also, the variation in younger groups (0-1 and 2-4 years old) is significantly larger compared to the older age groups (5-14 and 15+ years old).


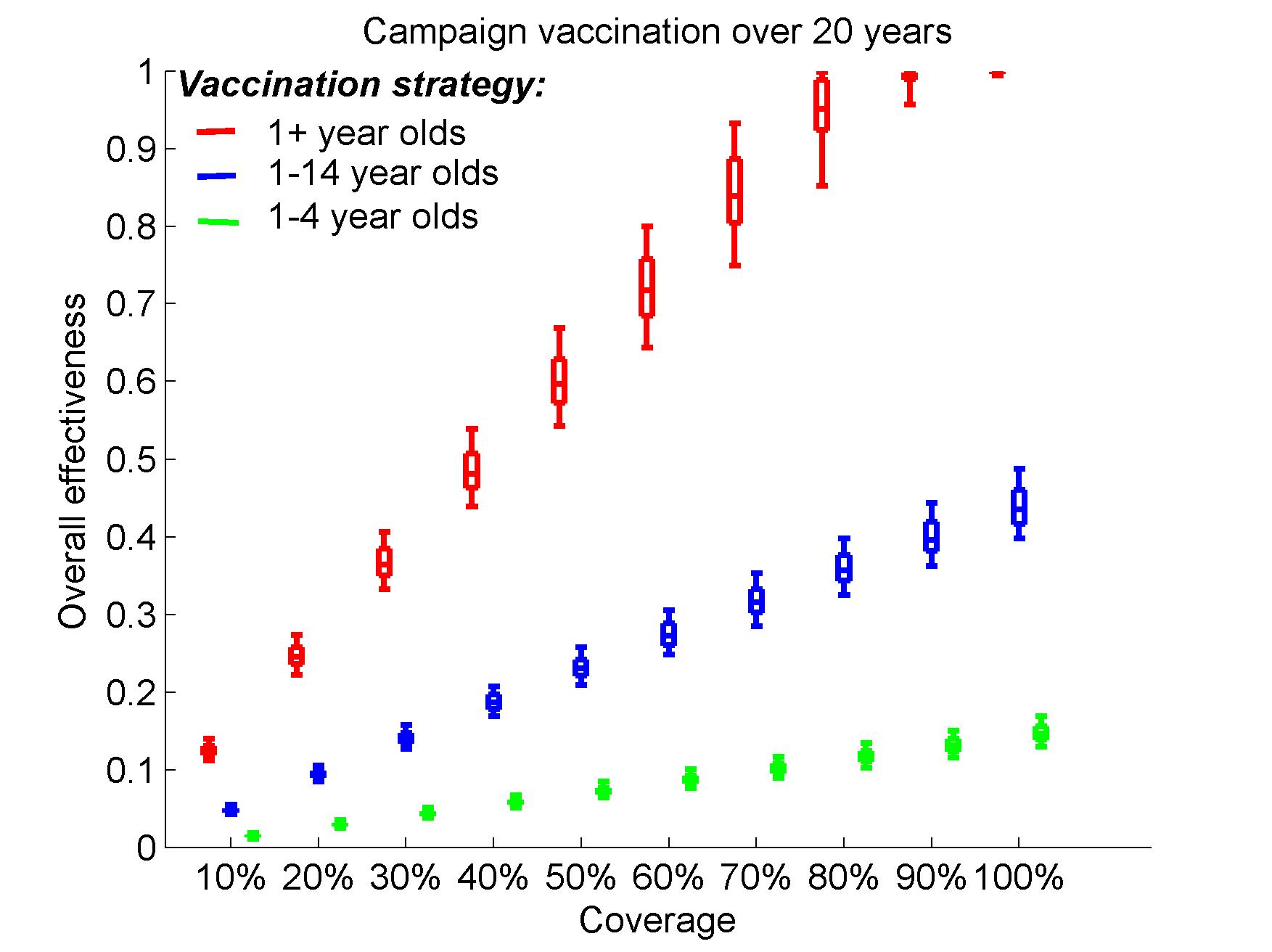

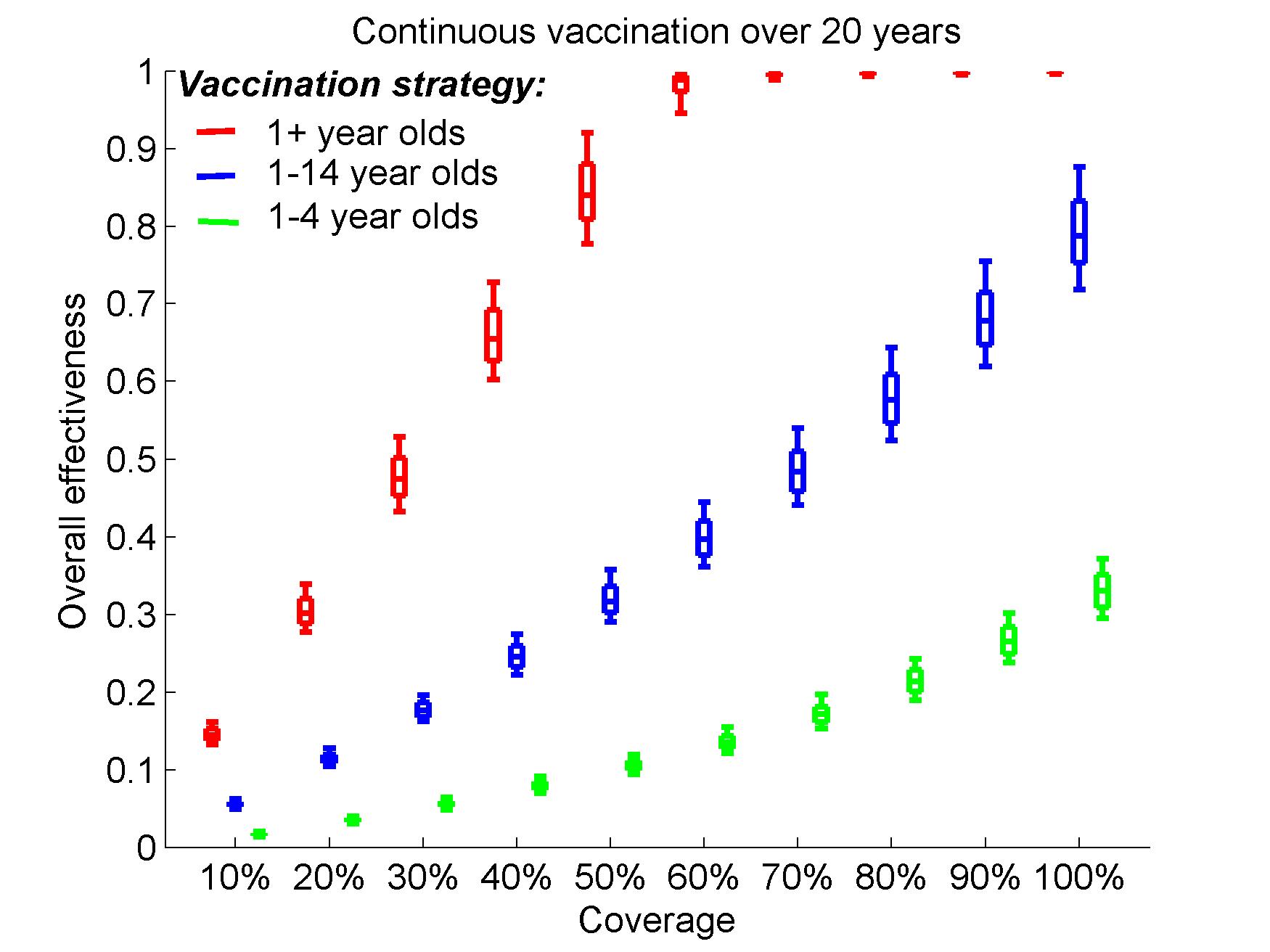


A)

B)

**Figure S9. Effectiveness of different vaccination strategies** for vaccine with 5 years of average protection **assuming different duration of natural immunity across age groups.** (1 year, 2 years, 3 years, and 5 years, respectively for age groups 0-1, 2-4, 5-14, and 15+ years old). Vaccine is less effective for children 1-4 years old (40% efficacy) than for older children and adults (65% efficacy) and 10% of the cholera cases are reported. Overall effectiveness of A) 5-year campaign vaccinations and B) continuous vaccinations over 20 years. The boxes represent the interquartile range and the whiskers cover 90% of the results from 100 simulations per scenario. Note that 90% and 60% coverage is enough for the campaign and continuous vaccination respectively to prevent 90% of the cholera cases which is a slightly more optimistic compared to the main scenario. Vaccinating all children (1-14 years old) continuously will prevent 79% of cholera cases which is similar to the fraction prevented when vaccinating 50% of the total vaccine-eligible population.
